# Guided and Unguided Internet-Based Treatment for Problematic Alcohol Use – A Randomized Controlled Pilot Trial

**DOI:** 10.1371/journal.pone.0157817

**Published:** 2016-07-06

**Authors:** Christopher Sundström, Mikael Gajecki, Magnus Johansson, Matthijs Blankers, Kristina Sinadinovic, Erik Stenlund-Gens, Anne H. Berman

**Affiliations:** 1 Department of Clinical Neuroscience, Centre for Psychiatry Research, Karolinska Institutet, Stockholm, Sweden; 2 Department of Public Health, Karolinska Institutet, Stockholm, Sweden; 3 Stockholm Center for Dependency Disorders, Stockholm, Sweden; 4 Arkin Mental Health Care, Amsterdam, The Netherlands; 5 Trimbos, Netherlands Institute of Mental Health and Addiction, Amsterdam, The Netherlands; 6 Department of Psychiatry, Academic Medical Centre, University of Amsterdam, Amsterdam, The Netherlands; 7 Department of Psychology, Stockholm University, Stockholm, Sweden; Hvidovre Hospital, DENMARK

## Abstract

**Background:**

The Internet has increasingly been studied as mode of delivery for interventions targeting problematic alcohol use. Most interventions have been fully automated, but some research suggests that adding counselor guidance may improve alcohol consumption outcomes.

**Methods:**

An eight-module Internet-based self-help program based on cognitive behavioral therapy (CBT) was tested among Internet help-seekers. Eighty participants with problematic alcohol use according to the Alcohol Use Disorders Identification Test (AUDIT; scores of ≥ 6 for women and ≥ 8 for men) were recruited online from an open access website and randomized into three different groups. All groups were offered the same self-help program, but participants in two of the three groups received Internet-based counselor guidance in addition to the self-help program. One of the guidance groups was given a choice between guidance via asynchronous text messages or synchronous text-based chat, while the other guidance group received counselor guidance via asynchronous text messages only.

**Results:**

In the choice group, 65% (13 of 20 participants) chose guidance via asynchronous text messages. At the 10-week post-treatment follow-up, an intention-to-treat (ITT) analysis showed that participants in the two guidance groups (choice and messages) reported significantly lower past week alcohol consumption compared to the group without guidance; 10.8 (SD = 12.1) versus 22.6 (SD = 18.4); *p* = 0.001; Cohen’s *d =* 0.77. Participants in both guidance groups reported significantly lower scores on the AUDIT at follow-up compared to the group without guidance, with a mean score of 14.4 (SD = 5.2) versus 18.2 (SD = 5.9); *p* = 0.003; Cohen’s *d =* 0.68. A higher proportion of participants in the guidance groups said that they would recommend the program compared to the group without guidance (81% for choice; 93% for messages versus 47% for self-help).

**Conclusion:**

Self-help programs for problematic alcohol use can be more effective in reducing alcohol consumption over a 10-week period when counselor guidance is added.

**Trial Registration:**

Clinicaltrials.gov NCT02384304

## Introduction

The World Health Organization estimates that alcohol is a causal factor in 5.9% of all global deaths and 5.1% of the global burden of disease [1]. However, no more than one out of three problem drinkers actually seeks help within the health care system, in what is often referred to as the treatment gap [2]. The Internet has long been regarded as having great potential for narrowing the treatment gap, since Internet-based interventions are accessible at any time, and may be perceived as more discreet and thus less stigmatizing than face-to-face interventions [3]. Internet-based interventions for problematic alcohol use have been studied for a number of years, both in brief and more extended formats. Internet-based screening and brief intervention (eSBI), the most extensively studied format, usually consists of a single session and mainly focuses on screening of current alcohol use and provision of personalized normative feedback to the user [4–6]. eSBIs have been shown to be effective in reducing alcohol consumption, for periods of up to 12 months [7], with an average effect size of 0.27 compared to diverse control groups such as waiting list, alcohol education and assessment only [8]. More extended Internet-based interventions are based on principles of cognitive behavioral therapy (CBT) and motivational interviewing [9–11] and are intended for continual use over a number of weeks. Compared to single-session interventions, effect sizes for more extended interventions were first shown to be somewhat larger (Hedges’ *g =* 0.61 compared to 0.27) [8], but a later meta-analysis showed no significant differences between shorter and more extended interventions [12].

One of the more consistent findings in research on Internet-based interventions for psychiatric disorders such as depression and anxiety is that receiving guidance from a counselor or coach when using an intervention has major positive implications for outcomes [13]. However, little research is available on the effects of guidance when delivering Internet-based interventions targeting problematic alcohol use. In one meta-analysis no significant differences were found between guided and unguided interventions, but the authors concluded that this may be due to the shortage of studies and that more studies with counselor guidance are warranted, preferably ones where a group receiving counselor guidance is explicitly compared to a group receiving no such guidance [12]. In the most recent systematic review, where the general conclusion was that e-interventions for alcohol use tend to produce small short-term benefits, studies with more intensive interventions that include human interaction were recommended, as these may produce more enduring benefits [14]. To our knowledge, only one study has investigated the effects of a full manualized Internet-based CBT self-help intervention, specifically comparing groups with and without counselor guidance. In this study, one group received the intervention with seven weekly chat sessions with a therapist, one group received the intervention as self-help, without chat sessions, and one group was put on a waiting list. The study reported a significant difference between guided and unguided self-help at six months post-randomization, in favor of the guided intervention. However, there was no difference between the two intervention forms at the three month follow-up, which occurred immediately after treatment [15].

If adding guidance to an Internet-based intervention leads to greater reductions in alcohol consumption, this could have important clinical implications for the field. In view of the lack of research on this issue, we sought to partially replicate the above-mentioned study [15]. As in the original study, we offered Internet help-seekers guided and unguided self-help. In the original study, guidance was offered to participants only via synchronous text-based chat, although Internet-based treatment has mostly been offered via asynchronous messages [16]. To assess the respective popularity of chat and messages, we added one trial arm in which participants could choose their preferred form of counselor guidance. Previous research has shown that giving study participants a choice of administration method can reduce attrition compared to a group not given such a choice [17].

### Specific research questions

This trial was a three-arm randomized controlled pilot trial where the primary aim was to evaluate the feasibility and preliminary effects of an eight-module Internet intervention for problematic alcohol use, with and without guidance. In terms of feasibility, we were interested in seeing to what extent participants would enroll in the study and find the intervention acceptable to engage with. In terms of preliminary effects, we investigated whether guidance might have any differential effect on alcohol consumption compared to the unguided group. Further, we wanted to explore levels of interest in two different forms of counselor guidance: asynchronous text messages and synchronous text-based chat, and also to explore whether offering a choice between these two forms of communication might influence attrition.

## Method

### Study design

This study used a three-arm design with randomized allocation (ratio asynchronous: synchronous: no guidance = 1:1:2) of Internet help-seekers reporting problematic alcohol use. Participants were recruited online through the Swedish Internet site Alkoholhjälpen (www.alkoholhjalpen.se), an open access website that provides information and a discussion forum to individuals seeking help for their alcohol consumption. The site has been publicly accessible since 2007 with approximately 500 visitors daily (for more details see [18]).

### Procedure

Between December 17, 2012 and January 4, 2013, a short information text about a study on Internet-based help for problematic alcohol use was available to visitors at the Alkoholhjalpen.se site. Interested visitors could click on a link leading to a study information web page stating that study participants would be randomly allocated to one of three groups, and that two of the groups would include counselor guidance. Information was also given about the handling of personal data. Individuals wishing to participate gave their informed consent by clicking on an icon at the bottom of the web page. They were then directed to a new web page where they were required to fill out gender, age, and web-based Swedish versions of the Alcohol Use Disorders Identification Test (AUDIT [19]) and the Drug Use Disorders Identification Test (DUDIT [20]). Those with an AUDIT score indicating at least hazardous use, i.e. ≥6 for women and ≥8 for men [21, 22], and who were at least 18 years of age were considered eligible for inclusion in the study. Participants were asked to provide a user name and password for secure access to the online intervention, as well as contact data in the form of an email address and phone number. A personal account was then created.

### Randomization

**Fig 1:**
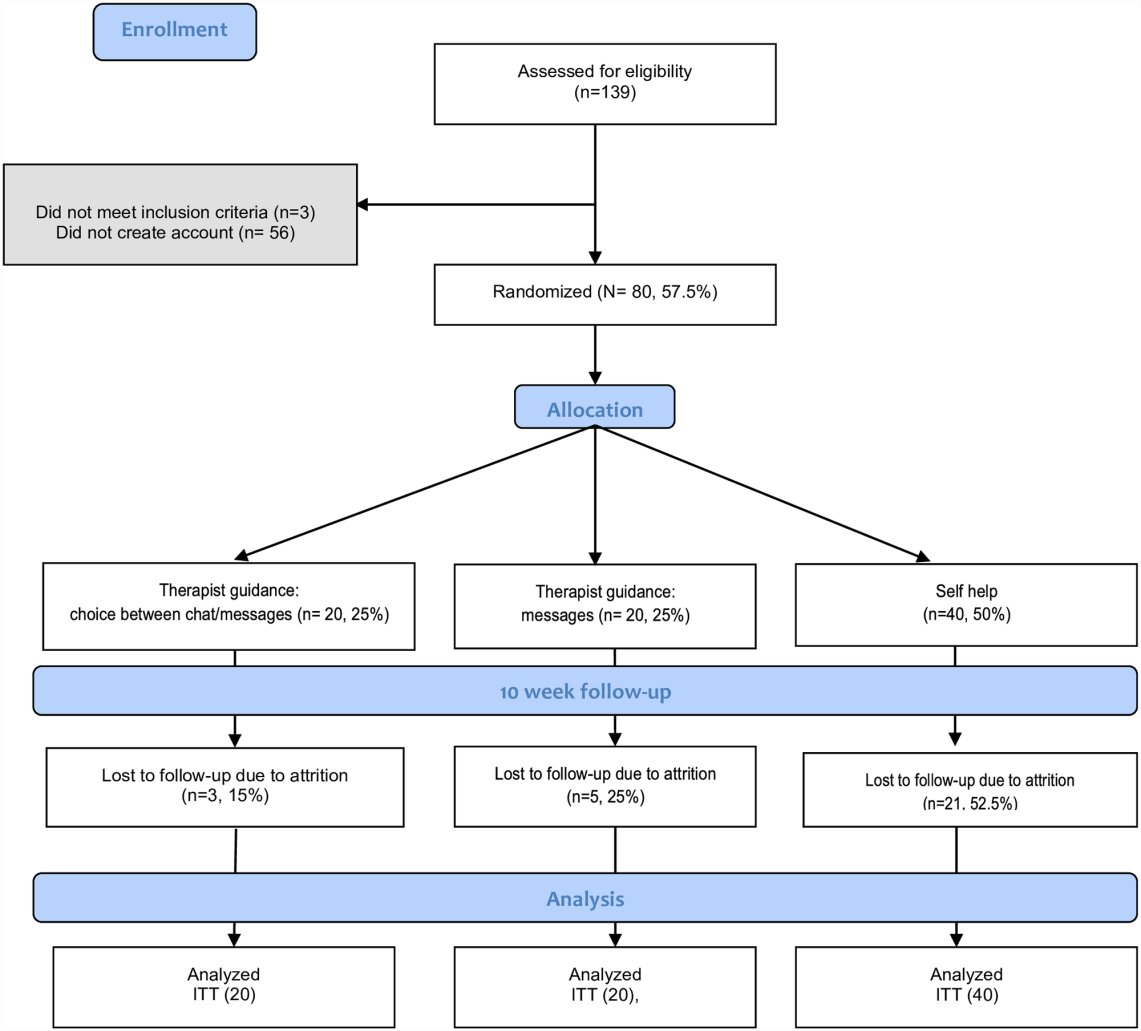
Flowchart showing enrollment procedure, randomization and analysis (ITT = Intention to Treat).

Following completion of the AUDIT and DUDIT questionnaires and creation of the personal account, eligible participants were randomized in blocks of 20, according to a fully automated and concealed procedure in the online platform. Participants were assigned to one of three groups: one receiving an Internet-based self-help program with counselor guidance via asynchronous messages; one receiving an Internet-based self-help program with counselor guidance offered through a choice between synchronous chat and asynchronous messages, and one receiving the Internet-based self-help program without counselor guidance. Recruitment was closed after 80 participants had been included (40 in the self-help group and 20 in each counselor guidance group). Participants filled in baseline questionnaires, described below under “Measures”, and then were informed about their allocation. Several of the authors (CS, MG, MJ, ESG and AHB) also acted as counselors and were thus not blind to participant allocation. Fig 1 summarizes the participant flow in the study.

### Internet-based treatment

Participants in all three groups had access to the same treatment program, a Swedish translation and adaptation of the Dutch program Therapy Alcohol Online, developed by the Jellinek Clinic [15, 23]. The program was delivered through a newly designed web portal secured with encrypted communication and protected with an individual user name and a password. The program consisted of eight online modules with 1–3 pages of reading material per module along with exercises where the participant can register answers and write texts pertaining to the exercises. The following modules were included in the program: Module 1: Analyzing advantages and disadvantages of drinking; Module 2: Setting an alcohol consumption goal (moderating or abstaining from drinking); Module 3: Learning self-control skills; Module 4: Identifying risk situations; Module 5: Managing craving, emotions, and social pressure (three separate modules, where the user was instructed to focus first on the one most relevant for him or her); Module 6: Developing a crisis plan; Module 10: Follow-up questionnaires and program evaluation questions (not part of the original Dutch program). Program content was made accessible to the user once a week, as described above. There was a three-week gap between weeks 6 and 10 to give the participant an opportunity to freely use any exercises in the program, as needed.

Each participant received an email every time new content was made accessible. During the treatment period, participants were strongly encouraged to regularly register craving as well as daily alcohol consumption. An electronic private diary was also available to participants; counselors did not have access to participant diary entries. All participants could access the self-help program whenever they wanted during the study as well as having access for an unlimited period following the end of the study.

### Groups

One group was given access to the self-help program with no counselor guidance (self-help group). Two groups received the self-help program with counselor guidance. One of the guidance groups received counselor guidance via secure text messages on the platform. There were no set guidelines for contact frequency. However, counselors aimed to answer participant messages within 48 hours, and could also answer at shorter intervals when deemed appropriate. Participants were automatically sent an email each time a message from the counselor had been sent to them from within the platform (Guidance: message group). Participants allocated to the second guidance group had access to the self-help program, and were asked before beginning the program to choose between either asynchronous guidance via secure text messages on the platform (as described in group 2) or synchronous counselor guidance entailing a live 40-minute text-based chat on the platform once a week at an agreed time (Guidance: choice group). Seven counselors guided the participants in the two guidance groups. All had basic training in MI and/or CBT-based psychotherapy. Supervision was given as needed by the senior author (AHB).

### Follow-up

One week after the last module had been made accessible, a timepoint 10 weeks after randomization, participants were emailed and asked to log into the system to complete the follow-up questionnaires. Participants who did not respond to the initial request and two e-mail reminders were contacted by telephone. The follow-up package consisted of the same questionnaires as at baseline as well as evaluation questions about Internet-based treatment (originally formulated by Postel and colleagues [24]) and questions about other forms of support and treatment for problematic alcohol use accessed by participants during the treatment period [25].

### Measures

#### Primary outcome measure

Timeline Follow Back (TLFB) is a method of measuring consumption of alcohol in terms of standard units over a specified length of time [26]. In the present study, participants were asked about their drinking during the preceding 7 days. TLFB delivered via computer has been found to yield data that correlate highly with paper and pencil [27]. Test-retest evaluation of a web-administered 7-day version of TLFB showed an intra-class correlation of 0.67, deemed fair [28].

#### Other measures

The Alcohol Use Disorders Identification Test (AUDIT) is a well-established and widely used 10-item instrument for measuring alcohol consumption and signs of harmful use and dependence related to alcohol consumption [29, 30]. The instrument renders a score between 0–40. A score of between 6 and 13 for women and between 8 and 15 for men is considered hazardous use [31]; a score of between 14 and 19 for women, and between 16 and 19 for men is considered harmful use. A score of 20 or above for both men and women is considered probable alcohol dependence [32]. The web-administered Swedish version has shown internal consistency reliability in terms of Cronbach’s α values at 0.80–0.93 [6, 17]. AUDIT-C refers to the first three questions of the AUDIT, that concern only the individual’s actual consumption [33].

The Drug Use Disorders Identification Test (DUDIT) is an 11-item questionnaire designed to assess patterns of drug consumption and drug-related problems The instrument renders a score between 0–44. A score between 8 and 24 is considered harmful use [34], and a score of 25 or above is considered probable drug dependence [35]. The DUDIT has shown good internal consistency reliability for a web-administered Swedish version at Cronbach’s α = 0.86 [17].

The Hospital Anxiety and Depression Scale (HADS) is a 14-item questionnaire consisting of two subscales used to identify anxiety (HADS-A subscale) and depression (HADS-D subscale) [36]. Each subscale has seven items and renders a total score of between 0 and 21. A score of 8 or above on any of these two subscales is considered to indicate a clinically relevant condition. Both subscales have demonstrated good internal consistency reliability: HADS-A (Cronbach’s α at 0.68–0.93; mean = 0.83) and HADS-D (Cronbach’s α at 0.67–0.90; mean = 0.82) [37]. Internet administration of HADS has been shown to yield similar results to paper-and-pen versions [38].

The Readiness to Change Questionnaire (RCQ) is an instrument for measuring the respondent’s motivation for change. The instrument contains 12 questions that, taken together, place the respondent either in the pre-contemplation, contemplation or action phase of the Trans-Theoretical model of change [39]. The Swedish version has yielded excellent internal consistency, where Cronbach’s α = 0.88 [40]. To the authors’ knowledge, there is no validation of RCQ for web administration.

The Readiness Ruler consists of a Visual Analogue Scale (VAS), where respondents are instructed to indicate their responses on a scale of 0–10 in relation to the statements: "I am not ready to change my drinking habits" (0) and "I am very ready to change my drinking habits" (10). The scale has been shown to have good predictive properties for reducing future alcohol consumption, measured after three months [41]. To the authors’ knowledge, there is no validation of the Readiness Ruler for web administration.

EuroQol-5 dimension (EQ-5D) is an assessment instrument for measuring quality of life. It consists of five items each covering one of five quality of life dimensions: mobility, self-care, usual activity, pain/discomfort and anxiety/depression. The item for each dimension is scored on a 1 to 5 Likert scale, and together they generate an index score between 0 and 1. In addition to the five items, the EQ-5D includes a VAS question on a scale of 0–100 regarding the respondent’s appraisal of his/her own current health status [42]. In a study in an HIV/AIDS population, the EQ-5D yielded a high Cronbach’s α of 0.85 for internal consistency [43]. We calculated the index score with the Crosswalk value sets, using the UK as a reference [44]. To the authors’ knowledge, there is no validation of EQ-5D for web administration.

The World Health Organization Quality of Life Scale-abbreviated version (WHOQOL-BREF) consists of 26 questions measuring quality of life on four domains: physical, psychological, social and environmental. The items are measured on a Likert scale of 1 to 5. Scores for each of the four domains are transformed into a score on a 0–100 scale. In a general population sample in Australia, scores were found to be around 70–75 on each domain scale [45]. The instrument has yielded fair to good results for internal consistency, with Cronbach’s α of 0.68–0.82 in multiple language versions [46]. A recent study validated different online versions of the instrument, and found that these generated the same Cronbachs’s alpha as previous paper-and-pencil versions [47].

### Sample size and statistical analyses

No power calculation was performed prior to the initiation of this study due to its pilot status, where the aim primarily was to test feasibility and, only secondarily, to test preliminary effect sizes. The sample size was set a priori to a total of 80 individuals, to obtain a minimum of 20 individuals in each of the counselor guidance groups and 40 in the self-help group. Analyses were first conducted comparing the three intervention groups. In order to answer the broader question of the general effect of counselor guidance on alcohol consumption, analyses were also conducted comparing two groups, where the two guidance groups were collapsed into one and compared to the self-help group. Descriptive statistics were used to describe baseline characteristics. To determine any baseline differences between the groups, one-way analysis of variance (ANOVA) and *t*-tests were used for continuous variables and Pearson’s chi-square test was used for categorical variables. Data were analyzed according to the intention to treat (ITT) principle. Missing follow-up data for the 80 participants were multiply imputed for TLFB, AUDIT, HADS, EQ5 and WHOQOL-BREF. The Amelia II package for R, version 3.0.3 (http://cran.r-project.org/web/packages/Amelia/) was used for multiple imputation [48], as it has been previously shown to suit TLFB data [49]. Five imputed data sets were created. Square root transformation on skewed count data (TLFB) was performed before the imputations. All statistical analyses were performed using IBM SPSS Statistics for MacOS X, Version 22 (IBM Corp, Armonk, NY, USA). Outcomes were analyzed with Generalized Estimating Equations (GEE) in a three (Groups: 1. Guidance choice; 2. Guidance messages; 3. Self-help) by two (Time: Baseline and 10-week follow-up) design and a two (Groups: 1. Guidance; 2. Self-help) by two (Time: Baseline and 10-week follow-up) design, using an unstructured working correlation matrix. For TLFB, a negative binomial model with log link was used [50]. For all other measures, a normal model was used. In GEE calculations, SPSS generates pooled means and standard errors, but does not generate significance testing for pooled data. In order to perform significance tests on outcome data, we used an online calculator, where we entered the pooled means and standard errors [51]. For the three-group analysis, we performed *F*-tests and for the two-group analysis, we performed *t*-tests.

### Ethics and trial registration

The study was approved by the Stockholm Regional Ethical Review Board (ref nr 2012/1761-31/5, November 8, 2012). The trial was registered retrospectively at Clinicaltrials.gov (NCT02384304). The authors confirm that all ongoing and related trials for this intervention are registered (NCT02283593; NCT02377726 and NCT 02645721).

## Results

### Descriptive characteristics at baseline

Eighty participants completed the AUDIT and were included in the study and randomly assigned to study arms, but only 77 of these completed the full baseline questionnaire battery. Of the participants, 60% were women and the mean age was 42.3 years, ranging from 18 to 74. One of the participants entered an extreme value at age of 527, which was adjusted to the average age of 55 for the descriptive analyses. The mean alcohol consumption during the week prior to recruitment was 29.4 standard units of alcohol. According to the RCQ, no participants were in the Precontemplation stage, 91% were in the Contemplation stage and 9% were in the Action stage (X_2_ = 4.945, p = 0.293). The mean rating of ‘readiness to change’ on the Readiness Ruler for all participants was 8.5 (SD = 1.8, F = 0.327, p = 0.723). Seven of the participants had scores of ≥ 1 on the DUDIT (m = 8.1; SD = 4.5). There were no significant differences between the three groups in any of the baseline characteristics. Baseline characteristics of the participants included in each of the three study groups are shown in Table 1, with 10-week follow-up data.

**Table 1:** Baseline and treatment (10-week) follow-up measures for the three study groups.

	Before treatment								After treatment								
	Guidance: choice	Guidance: messages	Both guidance groups combined	Self- help	3-group comparison		2-group comparison		Guidance: choice	Guidance: messages	Both guidance groups combined	Self-help	3-group comparison		2-group comparison		2-group effect size
**Female**	12 (60%)	15 (75%)	27 (67.5%)	21 (52.5%)													
**Age**	40.6 (11.8)	43.2 (9.8)	41.9 10.9)	42.7 (13.4)													
	M (SD)	M (SD)	M (SD)	M (SD)	**3-group** F-test	**3-group** p-value (F)	**2-group** t-test	**2-group** p-value (t)	M (SD)	M (SD)	M (SD)	M (SD)	**3-group** F-test	**3-group** p-value (F)	**2-group** t-test	**2-group** p-value (t)	Cohen’s D
**TLFB (drinks preceding 7 days)**	30.2 (20.3)	27.6 (16.3)	28.9 (18.2)	29.8 (15.4)	0.142	0.868	-0.235	0.815	11.6 (13.8)	10.0 (9.7)	10.8 (12.2)	22.6 (18.4)	6.454	0.003**	3.385	0.001**	0.77
**AUDIT**	22.0 (5.3)	20.1 (4.3)	21.00 (4.9)	22.2 (5.8)	1.140	0.325	-1.004	0.318	15.1 (5.7)	13.8 (4.7)	14.4 (5.2)	18.2 (5.9)	5.345	0.007**	3.037	0.003**	0.68
**AUDIT-C**	8.0 (1.9)	7.8 (1.3)	7.9 (1.6)	8.4 (2.0)	0.954	0.390	-1.345	0.182	5.9 (2.5)	5.1 (1.8)	5.5 (2.4)	6.4 (3.1)	1.861	0.163	1.525	0.131	0.34
**HAD-A**	11.3 (4.8)	10.9 (5.0)	11.1 (4.9)	11.4 (4.3)	0.059	0.942	-0.243	0.809	7.9 (4.1)	9.3 (4.5)	8.6 (4.7)	8.4 (8.8)	0.225	0.799	0.143	0.887	0.03
**HAD-D**	6.6 (4.5)	6.4 (4.6)	6.5 (4.5)	7.8 (4.0)	0.956	0.389	-1.378	0.172	4.5 (4.9)	4.6 (4.4)	4.6 (4.6)	5.9 (7.9)	0.461	0.633	0.914	0.364	0.20
**EQ5 index**	0.72 (0.17)	0.78 (0.13)	0.75 (0.15)	0.69 (0.17)	2.020	0.140	1.610	0.111	0.77 (0.17)	0.76 (0.22)	0.76 (0.20)	0.76 (0.19)	0.003	0.997	0.067	0.947	0.00
**EQ5 VAS**	59.2 (23.3)	56.7 (25.1)	58.0 (23.9)	53.0 (22.3)	0.487	0.616	0.939	0.351	74.9 (15.0)	72.3 (20.5)	73.6 (18.6)	65.0 (47.9)	0.657	0.521	1.067	0.289	0.24
**WHOQOL Phys**	59.8 (17.4)	63.2 (15.8)	61.5 (16.5)	56.7 (14.8)	1.113	0.334	1.341	0.184	68.5 (20.5)	71.1 (21.3)	69.8 (19.6)	68.1 (21.6)	0.150	0.861	0.368	0.714	0.08
**WHOQOL Psych**	47.4 (17.3)	50.0 (15.9)	48.7 (16.4)	40.5 (17.5)	2.322	0.105	2.115	0.038*	59.5 (21.8)	56.0 (21.5)	57.8 (22.6)	53.0 (29.2)	0.465	0.630	0.810	0.420	0.18
**WHOQOL Social**	52.6 (21.0)	46.1 (15.6)	49.3 (18.5)	47.0 (20.5)	0.674	0.513	0.523	0.602	58.0 (19.9)	55.0 (22.9)	56.5 (22.9)	57.5 (40.6)	0.053	0.949	0.136	0.892	0.03
**WHOQOL Envir**	70.2 (15.5)	68.8 (15.1)	69.5 (15.1)	63.0 (13.2)	2.061	0.135	2.013	0.048*	75.0 (16.0)	72.1 (17.8)	73.5 (17.6)	73.8 (21.7)	0.109	0.897	0.064	0.949	0.01

a. Due to significant baseline differences in WHOPsych and WHOEnvir, these variables were inserted as covariates in all follow-up statistical analyses

*p≤0.05;

**p≤0.01;

***p≤0.001

### Choice of counselor guidance

In the choice group, 35% (n = 7) chose to communicate with a counselor via chat and 65% (n = 13) chose messages. Two participants who chose chat switched to messages at their own request during the trial after a few chat sessions, and one participant did not participate in the chats at all. The other four participants received 3, 5, 7 and 8 chat sessions respectively during the treatment.

### Attrition

Attrition was defined as not participating in the follow-up. In total, 36.3% (n = 29) of the participants did not participate in the follow-up. In the choice group attrition was 15% (n = 3), two of whom had selected chat; in the messages only group attrition was 25% (n = 5) and in the self-help group it was 52.5% (n = 21). Baseline differences occurred between those who participated in the follow-up and those who did not: follow-up participants had higher physical wellbeing (61.9 vs 53.4; *t =* -2.30, *p =* 0.024) and higher environmental wellbeing (68.5 versus 61.7; *t =* -2.01, *p =* 0.048).

### Program use

**Fig 2:**
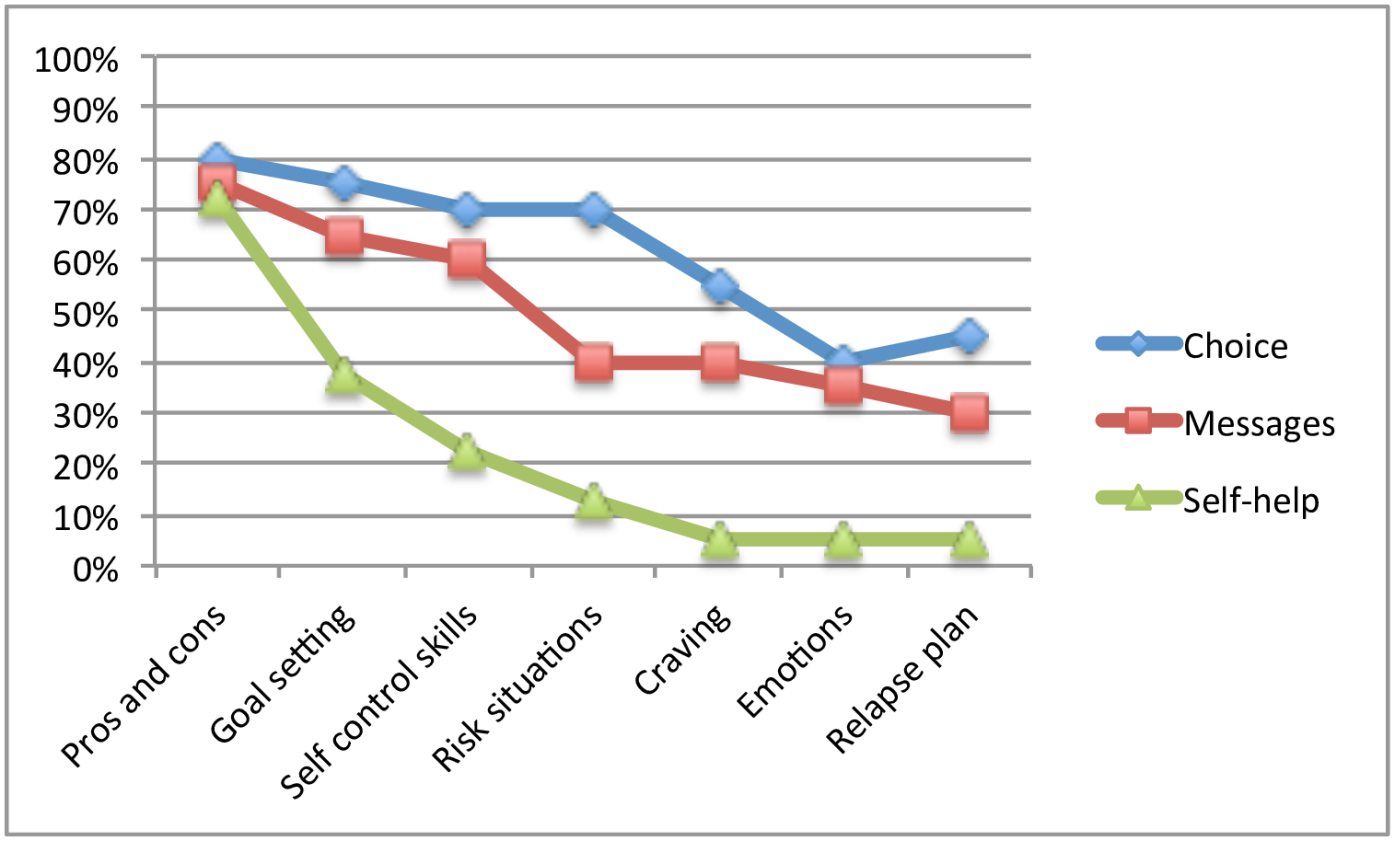
Proportion of participants completing each module in the two guidance groups (by choice or messages) and in the self-help group.

Out of 7 module exercises, the mean number completed (defined as the number of written and saved exercise entries) in the choice group was 4.4 (SD = 2.5), in the messages group it was 3.7 (SD = 2.7), and in the self-help group 1.5 (SD = 1.6). Fig 2 shows the proportion of participants completing each module.

The mean number of alcohol consumption registrations for the choice group was 19.1 (SD = 16.2) with a median of 10, for the messages group it was 24.9 (SD = 23.4) with a median of 13.5 and for the self-help group it was 3.8 (SD = 5.0) with a median of 1. Regarding use of the private diary, 30% (n = 6) of participants in the choice group used it, 40% (n = 8) participants did so in the choice group and 35% (n = 14) did so in the self-help group. Participants who chose messages in the choice group, or were allocated to messages, received a mean of 9.8 (SD = 16.6) and a median of 4 messages during the 10-week treatment period. An average of 5.8 chat sessions (SD = 2.2) were delivered to four participants.

### Evaluation questions and questions about access to other forms of help

At follow-up, participants were asked to evaluate the treatment from a participant perspective. In comparison to the self-help group, a larger proportion of participants who had received guidance experienced their contact with the program as personal, considered the program effective and would recommend it to others. In contrast, a larger proportion of the self-help group participants stated that they missed contact with a counselor and a lesser proportion considered the program effective. Nonetheless, both self-help and guidance groups indicated rather similar levels of insight into risk-related aspects of alcohol use. More detailed results are presented in Table 2.

**Table 2:** Participant evaluations of the treatment at follow-up.

	Guidance: Choice (n = 16)	Guidance: Messages (n = 15)	Self-help (n = 19)
**How did you like having contact with your counselor/treatment solely via the Internet? (scale 1–5) Was it…**			
**pleasant?**	4.3 (SD = 1.1)	4.2 (SD = 3.7)	3.7 (SD = 1.4)
**safe?**	4.3 (SD = 1.1)	4.4 (SD = 0.8)	3.7 (SD = 1.8)
**personal?**	3.7 (SD = 1.0)	3.7 (SD = 1.4)	2.2 (SD = 1.6)
**Did you miss (other kinds of) contact with your counselor, like through phone or face-to-face contact? Percent replying “yes”**	25%	20%	65%
**Do you consider the program an effective method for changing your drinking habit? Yes %**	75%	80%	29%
**Would you recommend the program to others? Yes %**	81%	93%	47%
**Did your willingness to seek professional help for your drinking problem in regular treatment increase during the program? Yes %**	31%	20%	29%
**Did you experience an increased insight into the following? Percent (%) answering “Yes”**			
**Your risk situations**	94%	87%	65%
**Your risk feelings**	75%	73%	71%
**Your risk thoughts**	88%	87%	59%
**The advantages of your alcohol consumption**	56%	73%	41%
**The disadvantages of your alcohol consumption**	94%	93%	76%

Participants were also asked about whether they had had access to other forms of help during the treatment period. In comparison to the self-help group, a larger proportion of guided participants had spoken to someone about their alcohol use. In contrast, a somewhat larger proportion of self-help group participants had used other forms of help on the Internet in comparison to the guided groups. See Table 3 for details.

**Table 3:** Participants’ reported use of other forms of help during the 10-week treatment.

	Guidance: Choice (n = 16)	Guidance: Messages (n = 15)	Self-help (n = 17)
**1. Had spoken to someone about their alcohol use**	81%	80%	47%
**2. Had used medication for their alcohol use**	13%	0%	12%
**3. Had used other help on the Internet**	6%	7%	29%
**4. Had used other help over the telephone**	0%	7%	0%
**5. Had used other help in writing**	13%	20%	18%

### Outcomes

#### Three-group analysis

In the follow-up questionnaires two individuals had multiple entries in the database. We decided to retain only first complete entry for these individuals as all individuals were meant to only provide one follow-up. As shown in Table 1, the choice group had reduced their alcohol consumption in the preceding week to 11.6 standard glasses (SD = 13.8), the messages group had reduced consumption to 10.0 standard glasses (SD = 9.7) and the self-help group had reduced it to 22.6 standard glasses (SD = 18.4). Both guidance groups decreased their preceding week alcohol consumption significantly more than the self-help group (*p =* 0.003). Furthermore, at follow-up the choice group had reduced their mean AUDIT score to 15.1 points (SD = 5.7), the messages group had reduced their mean AUDIT score to 13.8 points (SD = 4.7) and the self-help group had reduced their mean AUDIT score to 18.2 points (SD = 5.9). The score of the messages group was significantly lower than that of the self-help group (p = 0.007). Other secondary outcome data on anxiety and depression (HADS) and quality of life (EQ5 and WHOQOL-BREF) showed no significant differences between the groups. Data from RCQ are presented below only for the two guidance groups combined, compared to the self-help group.

#### Two-group analysis

When the two guidance groups were combined into one for the two-group analysis, baseline differences were found: those in the guidance group scored significantly lower on the two domains WHOQOL-Psychological Health and WHOQOL-Environment. These variables were inserted as covariates in all outcome analyses.

At follow-up, as shown in Table 1, the combined guidance group had reduced their alcohol consumption in the preceding week to 10.8 (SD = 12.1) standard glasses while the self-help group had reduced their alcohol consumption to 22.6 (SD = 18.4) standard glasses. The between-group difference was significant (p = 0.001) with a moderate to large effect size (Cohen’s *d* = 0.77). Furthermore, the combined guidance group had reduced their mean AUDIT score to 14.4 points (SD = 5.2) and the self-help group had reduced their mean AUDIT score to 18.2 points (SD = 5.9). This between-group difference was significant (p = 0.003), with a moderate effect size (Cohen’s *d* = 0.68). Secondary outcome data on anxiety and depression (HADS) and quality of life (EQ5 and WHOQOL-BREF) showed no significant differences between the groups. Regarding RCQ at follow-up, in the combined guidance group no participants were in the ‘precontemplation’ phase, 23.3% were in the ‘contemplation’ phase and 76.7% were in the ‘action’ phase. In the self-help group, no participants were in the ‘precontemplation’ phase, 41.2% were in the ‘contemplation’ phase and 58.8% were in the ‘action’ phase. There were no significant differences between the groups (Χ^2^ = 1,652; p = 0,199).

A central assumption when performing multiple imputation is that missing data has a MAR (missing at random) pattern; i.e., that the missingness is not related to the outcome measure that is missing. To investigate whether this assumption was legitimate, we conducted t-tests comparing baseline measures for those who participated in the follow-up with those who did not. The results showed that those who participated in the follow-up consistently rated their quality of life as significantly higher than those who did not participate. This was true in all four domains of WHOQOL-BREF as well as in EQ5D. There were no significant differences in any other measures (TLFB, AUDIT or HADS) (see Table 4).

**Table 4:** Baseline comparisons between participants in follow-up and those who did not participate in follow-up.

	Follow-up available	N	M (SD)	t	p
**TLFB**	Yes	49	31.1 (18.3)	1,205	0.232
	No	28	26.3 (13.3)		
**AUDIT**	Yes	49	21.0 (5.2)	-1,267	0.209
	No	31	22.5 (5.5)		
**HADS-A**	Yes	49	10.6 (4.8)	-1,617	0.110
	No	28	12.4 (4.0)		
**HADS-D**	Yes	49	6.6 (4.1)	-1,721	0.089
	No	28	8.3 (4.1)		
**EQ5D**	Yes	49	0.76 (0.13)	2,812	0.006*
	No	28	0.65 (0.19)		
**WHOQOL-Bref Physical**	Yes	49	63.0 (14.6)	3,056	0.003**
	No	28	52.2 (15.4)		
**WHOQOL-Bref Psych**	Yes	49	48.6 (16.8)	2,801	0.006*
	No	28	37,5 (16.4)		
**WHOQOL-Bref Social**	Yes	49	51.9 (17.6)	2,271	0.026*
	No	28	41.7 (21.2)		
**WHOQOL-Bref Envir**	Yes	49	69.6 (13.5)	2,846	0.006*
	No	28	60.3 (14.4)		

## Discussion

This randomized controlled pilot trial evaluated an eight-module Internet-based program offered to Internet help-seekers with problematic alcohol use. Participants were randomized to either one of two guidance groups or to an unguided self-help group. Participants who received guidance reported experiencing their treatment as more personal, more effective and more worthy of recommendation than participants in the self-help group. Guidance group participants also reported to a greater extent having spoken to someone about their alcohol use during the treatment than the self-help group, while the self-help group reported having used other forms of help on the Internet more than the guidance group. At the 10-week follow-up both alcohol-related measures (TLFB and AUDIT) were significantly lower for the two guidance groups combined, compared to the self-help group.

This study is the first in Sweden reporting on guided Internet-based treatment for problematic alcohol use. Recruitment to the study was surprisingly quick, implying a pressing demand among Internet help-seekers for this type of help. Further, it is one of only two studies explicitly exploring the effect of guidance for a self-help program for problematic alcohol use. Offering one group a choice between synchronous and asynchronous guidance is, to our knowledge, unique in this context and provides valuable insights into the preferences of participants in these kinds of studies. Furthermore, the reductions in alcohol use reported in the guidance group were surprisingly large, from an average of 29 standard drinks/week to an average of 11 standard drinks/week. This change seems meaningful from a clinical perspective, given that participants in the guidance group at follow-up had an alcohol consumption below the national recommendations for drinking for men (under 14 standard drinks a week), and close to the threshold for women (9 standard drinks a week) [52]. An important observation concerns the choice of chat. This alternative proved to be less popular in the choice group, where 7 out of 20 chose this alternative. During treatment, two of the participants who chose chat asked to switch to messages (and thus only received one chat session each), and out of the five participants who chose and received the chat alternative throughout the treatment, the number of chat sessions received by participants were limited to between 3 and 8, although 10 chat sessions were offered to all. Logistically, it proved hard to find time slots that fitted both participant and therapist, and this led to fewer contacts than initially planned.

The differences in alcohol-related outcomes between guidance and self-help in our study were similar to those found in the Blankers et al study [15]. However, in our study, these differences were significant already at the 10-week follow-up, whereas in the Blankers et al study, differences were significant at the 6-month follow-up, but not at the three-month follow-up. Although the overall design in our study was similar to the Blankers et al study, and addressed a similar population (both studies recruited through a public access website targeting individuals with alcohol problems) there are some differences between the two studies that impede direct comparison. The Blankers et al study used several exclusion criteria not used in the present study, which could have implications for interpretation when comparing the two samples. For example potential participants with severe co-morbidity and substantial drug use were excluded. Although our study participants did not report any substantial drug use, other psychiatric co-morbidities in our sample may have gone undetected, and we could thus hypothetically have included participants with severe psychiatric co-morbidity, a group generally considered more difficult to treat. However, we did include a questionnaire concerning depression and anxiety, which did not indicate a clinically relevant problem either at baseline or at follow-up. Even though the samples in the two studies were quite similar in gender, age and AUDIT scores, they differed in baseline number of drinks per week (TLFB), with a much higher drinking level in the Blankers’ et al study (about 44 drinks per week) compared to ours (about 29 drinks per week). This difference in quantity is quite large and could indicate that participants in our sample had less severe alcohol problems. However, the baseline AUDIT score was similar in both samples, indicating a similar severity level.

### Limitations

The fact that attrition is particularly high in e-health research is often referred to in the literature as the “Law of Attrition” [53]. Despite attrition being typical in this line of research, it is of course still a major limitation hampering interpretation of data. We managed attrition in our study statistically by performing multiple imputation. Imputation is always a second-hand option in analyses and is naturally never as reliable as ‘real’ data. Furthermore, it always comes with unverified assumptions such as data ‘missing at random’ (MAR). As noted in the text, we contend that our assumption that data in this study are MAR is legitimate, since the missing data did not seem to be related to alcohol consumption in itself, but to quality of life. This observation is interesting in its own right, and could indicate that quality of life is an important factor to be considered in future research. However, the fact that we do not know for certain what our data would have looked like had attrition been lower, is an important limitation of this study. Furthermore, with an attrition of 25% in the combined guidance group and 52.5% in the self-help group, we also had major differential attrition, a phenomenon generally considered a significant threat to internal validity. Differential attrition observed in a trial may be related to perceived efficacy or tolerability of that intervention [54]. The differences in attrition in this study might have been a result of participants’ awareness at recruitment that two groups would receive guidance and one group would not. Those who at recruitment were interested in receiving guidance, but were randomized to self-help, may have felt unmotivated to continue with the intervention after randomization. Differences in attrition between guidance and self-help could also have occurred during treatment because participants did not perceive the intervention as efficacious. Access to counselor support has been associated with lower attrition in a meta-analysis of Internet treatment for depression: self-help programs without guidance had attrition rates of 74% while guided programs had attrition rates of 28% [55]. Engagement with the program in the self-help group was much lower compared to the guidance groups. For example, only 5% of participants in the self-help group completed the last two modules, as compared to 37.5% in the combined guidance group (see Fig 2). Daily registrations of alcohol consumption were also significantly less frequent. Even though we can only speculate regarding weaker program engagement in the self-help group, those who participated in the post-treatment follow-up reported generally lower levels of satisfaction with the program in the self-help group compared to the guidance groups. The self-help group also sought other kinds of help on the Internet to a greater extent, possibly indicating dissatisfaction with the program.

A few additional limitations should be noted. One obvious limitation in this study was the lack of an untreated control group. Any causal effect of the intervention beyond the effects of counselor guidance was thus not possible to assess. We do not know if reductions in alcohol consumption observed in the self-help group would have been similar in a wait list control group. Furthermore, we only used a 10-week follow-up, so we cannot draw any conclusions about long-term effects. Another limitation often characterizing trials on problematic alcohol use is the lack of ‘objective’ outcome measures such as blood samples. For administrative reasons and due to lack of resources, including blood samples in the study was not possible. Additionally, although some user data were available (number of completed modules and registrations of alcohol consumption), other important user data such as the number of site visits, number of log-ins and length of time spent on the site, were not included in the database, making it difficult to fully assess engagement with the program as a whole. Finally, we used the AUDIT as a secondary outcome partly to allow for comparison to other, previous trials reporting AUDIT outcomes The use of the AUDIT as a secondary outcome measure at a 10-week follow-up may be considered a limitation, since seven of the 10 items in the AUDIT refer to events over the past 12 months. However, the AUDIT is frequently used in the literature for outcome evaluations of less than 12 months so this limitation is shared by many other studies [8].

### Future research

In a recent systematic review, electronic interventions for problematic alcohol use were found to reduce alcohol consumption by about one drink per week, but this effect was not maintained at 12-month follow-ups. Furthermore, no effects were found regarding outcomes more relevant for general health, such as consumption under recommended drinking limits and reducing binge drinking frequency [14]. To investigate the clinical potential of such interventions, the authors state that more intensive interventions should be considered, preferably ones that include some form of human support. Meta-analyses for depression and anxiety have indicated major advantages for guided over unguided Internet-based treatment [55, 56], but a first meta-analysis showed no significant advantages for guided programs for problematic alcohol use [12]. Results from the current pilot study, however, add to previously published research findings indicating that guidance does lead to greater reductions in alcohol consumption than unguided self-help when delivering an Internet-based intervention for problematic alcohol use [57]. In order to establish a more solid evidence base concerning the clinical potential of these interventions, future research should include outcome measures of mean alcohol consumption, and add outcome measurement of health-related effects such as consumption under recommended drinking limits and reduced binge drinking frequency. Including such outcome measures is a prerequisite for estimating the health care utility of such interventions. Future research should also, in adequately powered, controlled trials further explore guidance, preferably in comparison to unguided self-help, with longer follow-ups. We are currently conducting two such trials with several follow-ups (Trial registrations: clinicaltrials.gov NCT02377726 and clinicaltrials.gov NCT 02645721). Lastly, comparing guided Internet-based treatment for problematic alcohol use with ordinary face-to-face treatment is, so far, an unexplored area. Research on other psychiatric disorders suggests that guided Internet-based treatment has effects comparable to those of face-to-face therapy [58], and this could well be the case for problematic alcohol use. However, to our knowledge, no studies have been published which have tested this hypothesis. The time is ripe to more systematically investigate whether experiences from research on internet-based interventions for psychiatric conditions also hold true for the treatment of problematic alcohol use.

## Conclusion

Adding counselor guidance appears to augment the effectiveness of Internet-based self-help in reducing alcohol consumption, at least in the short term.

## Supporting Information

S1 CONSORT Checklist CONSORT Checklist. (DOC)

S1 Protocol Trial Protocol English version. (DOCX)

S2 Protocol Trial Protocol Swedish version. (PDF)
