# Supplementary material for: Guided and Unguided Internet-Based Treatment for Problematic Alcohol Use – A Randomized Controlled Pilot Trial
Source: PLoS One. 2016 Jul 6;11(7):e0157817. doi: 10.1371/journal.pone.0157817 (PMC4934861; doi:10.1371/journal.pone.0157817)
Supplement: S1 Protocol — (DOCX) [file pone.0157817.s002.docx]

Internet-based treatment for problematic alcohol use - with or without guidance?

A randomized controlled pilot study

Anne H Berman, Erik Stenlund-Gens, Christopher Sundström,

Magnus Johansson, Kristina Sinadinovic

**Background**

Problematic alcohol use is today the third largest cause of disease worldwide and one of the greatest challenges to public health, causing death and disability worldwide (WHO, 2009). From a global perspective, almost four per cent of all deaths worldwide are attributable to alcohol, which is more than, for example HIV / AIDS, violence or tuberculosis. Consumption of alcohol is regarded as the direct cause of 60 different types of diseases and injuries, and indirect cause to another 200. In Sweden alcohol consumption has increased since the mid-1990s. Although some decline has been seen in recent years, according to SoRAD estimates, consumption, is still almost 20 percent higher today than in the mid-1990s (H. Leifman & Ramstedt, Preliminary version 2009-11-11). In The Abuse commission's final report "Better response to abuse and dependence" (SOU 2011: 35, 2011) the investigator estimates that about 700 000 people in Sweden have a harmful use of alcohol and that 330 000 of these have an alcohol dependence. Regarding hazardous use of alcohol, a use not yet developed to harmful use or dependence, it is estimated that about a million Swedes have such hazardous use. In 2009, the Institute of Public Health reported that 18% of men and 10% of women had a hazardous alcohol use (Cleve Palm & Karlsson, 2009). Despite a relatively large part of the Swedish population having a problematic alcohol use, we know that most of them do not seek treatment (Blomqvist, Cunningham, Wallander, & Collins, 2007). A survey showed that only 18% of all the alcohol dependent people in the county had contact with the specialized addiction care (Andréasson, 2010). There seems to be a big group of people who need to change their drinking habits but who, for various reasons, do not come into contact with addiction care. In an opinion survey conducted on behalf of the National Institute of Public Health in 2009 78% of respondents stated that they would use the Internet to obtain information about alcohol and other drugs, and focus group interviews with risk consumers of alcohol has shown that the help people primarily want for their problematic use consists of reliable information, preferably in the form of web-based test. Studies on Swedish Internet-based self-test of problematic alcohol and drug use show that they are an effective way to reach both those with problematic use and groups less accessible to healthcare, such as women and young adults (Sinadinovic, Berman, Hasson & Wennberg, 2010; Sjölund, 2007).

In a comparison with the situation in the 1990s, alcohol consumption in Sweden has increased faster among women than among men (CAN, 2009) and a population screening in 2008 revealed no notable differences in the prevalence of problematic alcohol use between women and men, (a total of 20% of women and 22% of men had a problematic alcohol use). Problematic drug use was more common among women - 3.8% - than 1.8% among men (Sinadinovic, Wennberg, & Berman, 2011. The gender distribution within specialized addiction care in Stockholm County shows that only 33% of patients in 2010 were female and 67% male (A. Leifman, 2011). Similar figures are reported in the Netherlands (Postel, de Haan, best Huurne, Becker, & de Jong, 2011). This suggests that it is particularly difficult for addiction services to reach women with problematic use of alcohol and drugs. Meanwhile, earlier research has shown that women are particularly attracted to internet based interventions and that, when it comes to the use of such services, at least 50% are women (Koski-Jännes, Cunningham, Tolonen, and Botha, 2007; Postel et al., 2011; Sinadinovic, Berman et al., 2010). Delivering interventions for problematic alcohol and drug use over the Internet should be suitable for Swedish conditions where 85% of the Swedish population use the Internet: Sweden is one of the countries in the world where Internet use is highest and most widespread in society; additionally current Swedish research suggests that Internet-based interventions for problematic alcohol and drug use might appeal to users in the Swedish population (Sinadinovic, 2012). A Canadian study has also shown that the majority of people with problematic alcohol and/or drug use use the Internet, even to a greater extent than those without such problematic use (Cunningham, Selby, Kypri, & Humphreys, 2006).

To date, treatment for problematic alcohol use over the internet has been tested in Sweden only in self help format (without therapist support), where help seekers on the internet were randomly assigned to eScreen.se (screening site), alkoholhjalpen.se (self-help modules) or a control group that completed questionnaires; follow-up was done after 3, 6 and 12 months (Sinadinovic, Wennberg, Johansson, & Berman, 2012). Alkoholhjälpen.se yielded better effect for those who had a little less severe problems. Of those who used eScreen.se 70% went on to talk to someone about their problems, compared with 49% from Alkoholhjälpen and 56% for the control group. These results are consistent with the overall positive results of the studies included in the review articles and meta-analyzes (Newman, Szkodny, Llera, & Przeworski, 2010; Rooke, Thorsteinsson, Karpin, Copeland, & Allsop, 2010).

**Purpose**

The general purpose of the study is to perform a randomized pilot study testing a Swedish version of an existing Dutch e-health program, *Therapy Online (TO),* for the treatment of problematic alcohol use. The program has demonstrated good results in a randomized controlled study (Blankers, Koeter & Schippers, 2011).

Specific issues of the pilot study are three:

1. In previous research, our research team offered 1000 people, out of a random population sample, a choice between answering questions about alcohol and drug use via Interactive Voice Response (IVR, automated telephony) or the Internet. In the same study 2000 people were offered to answer questions via IVR, and an additional 2,000 people answered the questions via the Internet (web forms). It turned out that the group receiving a choice between IVR and the Internet participated to a significantly greater extent in the study of the response rate (Sinadinovic et al., 2011). In this study we want to examine whether the possibility to opt for email and instant messaging provides better retention in the study. In order to answer this question grup 1 ***consists of*** 20 individuals who get to choose *whether they* want to have contact with a therapist through synchronous chat sessions or asynchronous e-mail messages.
2. In the program developed by the Jellinek Clinic in Amsterdam (where the evaluation made by Blankers et al., 2011) seven 40-minute chat sessions in real time were delivered to participants. In Sweden, however, internet treatment with therapist support for patients with depression and anxiety diagnoses is given via asynchronous messages (internetpsykiatri.se in the Stockholm County Council). This delivery method is more flexible for both finishers treated, and also cheaper to the caregiver. We are interested in examining the extent to which it works to offer the TO with therapist support in the form of written messages (corresponding email). To answer this question ***group 2 will consist of 20*** individuals who are randomized to Therapy Online programwith therapist support in the form of asynchronous text messages.
3. The study published by Blankers et al. (2011) consisted of three arms: TO with therapist support in the form of seven 40-minute chat sessions in real time, TO without therapist support and a control group in the form of a waiting list. In this pilot study, we want to partially replicate Blankers et al. study and compare treatment outcomes for TO with therapist support, with the outcome of the TO without therapist support. In order to answer this question 40 individuals will be randomized into ***group 3 to receive*** TO without therapist support. Answering this question will be generated after the data analyzes where groups 1 and 2 will be compared with group 3.

**Design**

As stated above the study is a randomized, controlled pilot study [1] with three arms:

1. TO with therapist support, choice between synchronous chat or asynchronous messaging, *n* = 20
2. TO with therapist support, asynchronous *messages,* n = 20
3. *TO without therapist support = 40*

Measurement based questionnaires (see below) occurs at recruitment to the study (T1) and at the completion of the study 10 weeks after recruitment (T2). See Figure 1 for the flow chart.

**Materials**

*The treatment program TO*

The treatment used in this study is a Swedish translation of a Dutch program that has shown very good outcomes in a randomized controlled study, where the program was compared to a self-help variation without therapist support (Blankers et al., 2011). The program contains eight modules focusing on relapse prevention (Saxon & Wirbing, 2004).

*Figure 1.* Three-arm pilot study of Internet-based treatment for problematic alcohol use with and without dealing with support

*Outcome Measure*

AUDIT (Alcohol Use Disorders Identification Test) (Annex 5.1)   is a well established and widely used instrument for assessing alcohol consumption (Bergman & Källmén, 2002; Saunders, Aasland, Babor, De La Fuente, & Grant, 1993). It consists of 10 questions answered 0-4 with a final score between 0 and 40. The result is then divided into hazardous use, abuse and dependence. Limit the risk of abuse required for men at 8 points or more, for women at 6 points or more. There is also a short version of the instrument, the AUDIT-C ("consumption"), which contains only the first three questions and is used to map the current situation. On some occasions it may be expedient to use one single query to get a picture of someone's drinking. For these cases, question 3 concerning high episodic drinking is the one that is best correlated with the total score (Berman, Wennberg, & Källmén, 2012).

 DUDIT (Drug Use Disorders Identification Test) (Appendix 5.2)   is a questionnaire designed to find out drug consumption and drug-related problems of various kinds.   The test consists of 11 questions that are scored between 0 and 4, giving a final score of 44 points. The result of the test is divided into hazardous use, abuse and dependence. The test has been shown to identify problematic use in studies. As for the AUDIT, there is a shorter version of DUDIT, DUDIT C, where only the first four questions dealing with consumption included. This version of the test, however, is unfortunately not enough studied. What you can infer is that the limit of 1 point for hazardous use applies to both DUDIT C as well as for DUDIT. (Berman, Bergman, Palmstierna, & Schlyter, 2005; Berman et al., 2012).

TLFB (Timeline Follow Back) (Appendix 5.3) is an instrument for measuring the consumption of alcohol or drugs over time and has been used successfully in the periods up to 12 months back (Sobell & Sobell, 1992). There have also been attempts to use the method over a period of 24 months with mixed results (Day, Collins, Degenhardt, Thetford, & Maher, 2004). Sobell and Sobell (1992) generally suggest the help of a calendar chart during the time drinking should be assessed. Besides the calendar, you can use a written support to convert the amount of alcohol consumed to standard glass. Furthermore seeking man during the time period to identify so-called key dates (birthdays, holidays, weekends) and periods when drunk or abstention from alcohol. It then uses the key dates and periods as so-called anchor points to find out how much you drank then. One recommendation is to initially identify upper and lower limit for alcohol consumption during the period so that you know in which range you move. In the present study, we ask participants about their drinking during the last 7 days, as in the study conducted by Blankers et al. (2011). It is worth noting that no clear support existed for longer periods of study gives more reliable results.

RCQ (Readiness to Change Questionnaire) (Appendix 5.4) is a test developed to measure the motivation to change (Forsberg, Ekman, Halldin, & Rönnberg, 2004; Role Nick, Heather, Gold, & Hall, 1992). The test consists of 12 questions and follow DiClemente and Prochaska model of "stages of change" which describes the process when a person tries to solve its problems of dependence (Prochaska & DiClemente, 1986). In a recently published doctoral thesis psychiatric patients who changed their drinking habits after a short intervention from the beginning changed their habits to a greater extent than those who did not receive an intervention (Gordh, 2012). Questions have been raised about RCQs ability to predict outcomes (Forsberg et al., 2004).

VAS measures of motivation (Readiness Ruler) (Appendix 5. 4.1) consists of a scale where the respondent on a line places its response between two answers, in this case the statement *"I'm not ready to change my drinking habits" and "I'm very much ready to change my drinking habits."* High performance on the scale, especially in the upper tertiary, correlates with lower levels of drinking during follow-up after three months (Bertholet, Cheng Palfai, Samet, & Saitz 2009).

WHOQOL-BREF (Appendix 5.5) is a test to assess quality of life developed by the World Health Organisation. It consists of 26 questions that are scored from 1 to 5. The test is a short version of a longer WHOQOL-100. WHOQOL-BREF correlates strongly with WHOQOL-100 on all dimensions excluding that social (O'Carroll, Smith, Couston, Cossar, & Hayes, 2000). The test has been used in extensive research since it was introduced and keeps good cultural validity (Skevington, Lotfy, & O'Connell, 2004).

EQ-5D -5 L (Appendix 5.6) is an assessment instrument for quality of life that consists of 5 questions with five possible answers each. The test also includes a Visual Analogue Scale (VAS) to ask where the respondent is invited to mark their health on a graduated line from 0 to 100 (Rabin & Charro, 2001). The test is available in two editions: first, the EQ-5D 3L where each question has three possible answers per question, and the EQ-5D-5L with five answer options per question. In recent years evidering retained the five dimensions but all of a response alternatives were expanded from three to five (Herdman et al., 2011). This instrument has been used in various clinical populations and may be less sensitive to the quality of life in non-clinical populations (the Willige, Wiersma, Nienhuis, and Jenner, 2005), consequently the EQ-5D 5L and WHOQOL-BREF used in this study.

HADS (Hospital Anxiety and Depression Scale) (Appendix 5.7) is a questionnaire commonly used in health care to identify anxiety and depression. The form consists of a total of 14 questions, seven for anxiety and depression each, and scored 0-3. Maximum points for anxiety and depression is 21 and a result of 15 points or more suggests that further treatment measures should be initiated. The tool has in studies been compared to BDI (The Beck Depression Inventory) and STAI (Stait-Trait Anxiety Inventory) and has shown good psychometric properties, both with respect to the correlation with the BDI and STAI, to internal consistency and stability, and in test-retest (Michopoulos et al. , 2008).

SRS (Session Rating Scale) (Appendix 5.8.1 for group 1 & 2 and 5.8.2 for Group 3) is a tool to measure the alliance. It can be administered after each session or module. It is a short test where the respondent is asked to consider the extent to which he or she agrees with four different statements; *"I* felt *heard, understood and respected." "We worked with or talked about it that I wanted to work with and talk about."* " *The way we worked in suits me fine.* "And" *Today's talks suited me perfectly.* ". Grading is done on a VAS-scale (Visual Analogue Scale) with the above statements on the one hand and the claims formulated as negatives of the other. SRS is clearly analogous to the longer test designed to measure the same thing, both in terms of validity and reliability (Campbell & Hemsley, 2009).

Follow-up questions (Appendix 5.9.1 Group 1 & 2 and 5.9.2 of Group 3) will investigate how participants experienced the Internet treatment; if they missed having telephone or physical contact, if they experienced the treatment as effective and whether they would recommend the treatment to anyone. The answers are sometimes graded, sometimes formulated as "Yes," no "and" do not know " (Postel et al, 2011).

  Other forms of treatment (Appendix 5.10)  This short questionnaire developed by the research group contains three questions relating to whether the participant used other measures of help besides the Internet treatment to manage his or her problem, including questions about medication and whether the person had contact with other responders - both self-help, telephone or customary care. By controlling for other factors, it becomes easier to assess the impact of the completed treatment (Sinadinovic et al, 2012; Sinadinovic, Johansson, Wennberg, & Berman, 2010).

**Participants**

A total of 80 people seeking help via the Internet will be recruited (see below for the recruitment procedure). All information and informed consent will be done on the Internet-based platform in line with previous studies that we have conducted. Individuals who are 18 years or older with at least risky use of alcohol (≥6 p for women and ≥ 8 p for men) on the AUDIT included in the study. Furthermore, we will inform the participants that it is important that they intend to be available via the Internet for study within the next 10 weeks of the study. Persons under 18 and / or without risky use of alcohol are excluded. Persons who receive over 15 on the estimates of anxiety and depression the HADS, those who get a result that is two standard deviations or more below average in any of the instruments that measure quality of life (WHOQOL-BREF and the EQ-5D) may participate in the study but are recommended after 10 weeks to seek psychiatric help. Participants at the end of the study, scoring higher than 20 on the AUDIT will receive a recommendation to turn to specialized addiction treatment or addiction treatment provided by social services. Participants who, according to the Drug Use Disorders Identification Test (DUDIT), have an active drug use in parallel with the use of alcohol will be allowed to participate in the study, but if continuing drug use at the end of the study, they will receive a recommendation to above apply to the specialized addiction care or social services to continue help. Personal advice on this can be given if the person contacts the research team. Contact details will be available when logging on the personal user account, the treatment site. We expect that a smaller proportion of participants in the study will show signs of problematic drug use in parallel with problematic alcohol use. One population survey of drug problems from 2008, where DUDIT questionnaire was used, shows that the risk of drug consumption in 2% of the Swedish population and the harmful use of 2.8% (Sinadinovic et al., 2011).

**Procedure**

*Recruitment*

Recruitment for the study will be done through an ad posted on Alcohol Help [(www.alkoholhjalpen.se).](https://translate.google.com/translate?hl=sv&prev=_t&sl=sv&tl=en&u=http://www.alkoholhjalpen.se/) If recruitment in this way proves to be slow, ads on the Internet will be used. The advertisement informs that there is a study on the treatment of alcohol problems currently taking place and that there is the opportunity to participate. Those who show interest in participating must take part of the informed consent (see Appendix 4 .1) and specify gender and age, and fill in the AUDIT - form. If the inclusion criteria for age and / or hazardous use of alcohol is not met, the individual will be informed that the hazardous use of alcohol is not present in the answers, and that he / she therefore does not belong to the target group of the study (Annex 4.1. 1 .1). If the inclusion criteria are met, the participant receives information on this and creates a user account (Annex 4.1.1.2). Then the other questionnaires will be answered (see above under Materials). The randomization to the three study arms is fully automated. Participants may then immediately access the group via their own user account (Annex 4.1. 2-4). Participants will get access to a new module every week during the first 7 weeks. Then there is a break in treatment for three weeks. Then the participants get access to a finishing module. After completion of treatment, participants fill in outcome measurements again and processing issues and follow-up questions (Annexes 5.1-5.10).

*Treatment*

Participants in Group 1 who choose to have therapist support through chat, will receive this 40 minutes / week at a predetermined time. Chat for one will be in a private "chat room," on the treatment site they have access to through the personal user account. Participants in Group 1 who choose contact via messages, as well as participants in Group 2 will have access to therapist support through written messages once a week. The messages will be available through the treatment site. Participants will receive regular e-mail messages when messages from the therapist are posted on the treatment site. Participants in Group 3 will work on their own, without therapist support during treatment. These, however, if necessary, will be able to ask questions of the research team.

Participants in all groups will have a reminder sent to them via regular e-mail once a week when the new module is available, when messages are available, reminders of time for chat, for relevance, and so on. In case of no contact, reminders via email will be sent. Should the participant's mood considerably worsen during treatment so that the he or she cannot or should not continue treatment, they will be offered personal contact with a licensed therapist from the research group for communication about a health care contact that can suit the participant's current needs. All e-therapists have at least basic training in psychotherapy. During the course of treatment continuous internal supervision will be held once a week at the beginning of the study and then at least once every two weeks to discuss experiences and knowledge.

**Statistics**

The results will be reported in anonymised form and statistical group level. Descriptive statistics for study participants' properties based on battery of questions will be presented, as well as analyses of the differences between the groups in the outcome. The primary measure of alcohol consumption is the AUDIT-C and TLFB. Secondary measures are other issues relating to anxiety, depression and quality of life. The analyses will be checked for motivation for treatment (Annex 5.4), other treatment during the study (treatment issues, Annex 5.10). Otherwise, additional results of the working alliance (Annex 5.8) and the platform's usability (Annex 5.9) will be presented.

This study is a pilot study in which the primary objective is to generate information and knowledge about various forms of Internet mediated therapist support. Since this is a pilot study we expect information about the feasibility and direction of outcomes (probably no significant differences). This is the first time that internet treatment with therapist support is tested in Sweden and the launch fits well with the National Board of Health guidance on disease prevention, where web-based treatment for problematic alcohol use is highlighted as an evidence-based action and which emphasizes that care must be equitable and accessible to all (National Board of Health, 2012).

National Board guidelines further states that care should be constructed as a so-called *stepped Care* model where light interventions are considered first, followed by more extensive if the first intervention would not have the desired effect. Today there are first steps in such a model by eScreen.se (Sinadinovic, 2012) and Alkoholhjalpen.se (Sinadinovic et al., 2012) and the Alcohol Line (Damström-Thakker, personal communication, 2012). Internet treatment is needed to provide a further step in the model, when the self-screening (eScreen.se) or work with self-help (alkoholhjalpen.se) or telephone (Alcohol line) does not lead to reduced or eliminated drinking. The increased accessibility to treatment via the Internet could potentially treatment more accessible to people who live in rural areas. Since only 20% of people with alcohol dependence seek help through addiction services Internet-based treatment could also reach the large proportion of people regardless of residence do not seek help, possibly because of the risk of stigmatization (Blomqvist et al., 2007). Internet based treatment with therapist support is in the current only an option for a few psychiatric diagnoses, but when it comes to substance abuse problems, there is no such option yet. This study is an important step in making such treatment available in Sweden.

**References:**

Andreasson, S. (2010). New development of addiction care in Stockholm County - Reporting of survey and proposals, Preliminary version 2010-07-01. Stockholm: Swedish National Institute of Public Health

Bergman, H., & Källmén, H. (2002). Alcohol Use Among Swedes and a psychometric evaluation of the Alcohol Use Disorders Identification Test. *Alcohol & Alcoholism, 37,* 245-251.

Berman, AH, Bergman, H., Palmstierna, T., & Schlyter, F. (2005). Evaluation of the Drug Use Disorders Identification Test (DUDIT) in Criminal Justice and Detoxification Settings and in a Swedish Population Sample. *European Addiction Research, 11,* 22-31.

Berman, AH, Wennberg, P., & Källmén, H. (2012). *AUDIT and DUDIT - identify problems with alcohol and drugs [Audit and DUDIT - Identifying problematic alcohol and drug use].* Stockholm: Gothia publishers.

Bertholet, N. Cheng, DM, Palfai, TP, Samet JH, & Saitz, R. (2009). Does readiness to change predictor Subsequent alcohol consumption in medical inpatients with unhealthy Alcohol Use? *Addictive Behaviors, 34* (8), 636-640. doi: 10.1016 / j.addbeh.2009.03.034

Blankers, M., Koeter, MWJ, & Schippers, G. (2011). Internet Therapy versus Internet Self-help versus No Treatment for Problematic Alcohol Use. *Journal of Consulting and Clinical Psychology, 79* (3), 330-341.

Blomqvist, J., Cunningham, J., Wallander, L., & Collin, L. (2007). To improve their drinking habits - on different patterns of change and what the values mean. Stockholm: SoRAD.

Campbell, A., & Hemsley, S. (2009). Outcome Rating Scale and Session Rating Scale in psychological practice: Clinical utility of ultra-brief Measures. *Clinical psychologist, 13* (1), 1-9. doi: 10.1080 / 13284200802676391

CAN. (2009). Drug Trends in Sweden 2009 report 117, [http://www.can.se/documents/CAN/Rapporter/rapportserie/can-rapportserie-117-drogutvecklingen-i-sverige-2009.pdf](https://translate.google.com/translate?hl=sv&prev=_t&sl=sv&tl=en&u=http://www.can.se/documents/CAN/Rapporter/rapportserie/can-rapportserie-117-drogutvecklingen-i-sverige-2009.pdf) . Stockholm: Swedish Council for Information on Alcohol and Other Drugs.

Cleve Palm, J., & Karlsson, A.-S. (2009). Health on equal terms - Results from the National Public Health Survey 2009. Östersund: Swedish National Institute of Public Health.

Cunningham, JA, Selby, PL, Kypri, K., & Humphreys, NC (2006). Access to the Internet Among drinkers, smokers and illicit drug users: Is it a barrier to the commission of the intervention on the World Wide Web? *Medical Informatics and the Internet in Medicine, 31* (1), 53-58.

Day, C. Collins, L. Degenhardt, L., Thetford, C., & Maher, L. (2004). Reliability of heroin users' reports of drug use behavior using a 24 month timeline follow-back technique to Assess the Impact of the Australian heroin shortage. *Addiction Research & Theory, 12* (5), 433-443. doi: doi: 10.1080 / 16066350410001713231

the Willige, G. v., Wiersma, D., Nienhuis, FJ, & Jenner, JA (2005). Changes in quality of life in chronic psychiatric patient: A comparison between the EuroQol (EQ-5D) and WHOQoL. *Quality of Life Research, 14* (2), 441-451. doi: 10.1007 / s11136-004-0689-y

Forsberg, L. Ekman, S., Halldin, J., & Rönnberg, S. (2004). The readiness to change questionnaire: Reliability and validity of a Swedish version and a comparison of the scoring methods *the British Journal of Health* Psychology, 335-346.

Gordh, CN (2012). *Alcohol Use and Secondary Prevention in Psychiatric Care.* (PhD), Uppsala University, Uppsala.

Herdman, M., Gudex, C., Lloyd, A. Janssen, MF, Kind, P., Parkin, D.,. . . Badia, X. (2011). Development and preliminary testing of the new five-level version of the EQ-5D (EQ-5D-5L). *Quality of Life Research, 20* (10), 1727-1736. doi: 10.1007 / s11136-011-9903-x

Koski-Jännes, A., Cunningham, J., Tolonen, K., & Botha, H. (2007). Internet-based self-assesment of drinking - 3 month follow-up data. *Addictive Behaviors,* 32, 533-542.

Leifman, A. (2011). [Excerpt from VAL-database].

Leifman, H., & Ramstedt, M. (Preliminary version 2009-11-11). Swedish people's drinking habits in recent years, with the focus on the period 2004-2009. Stockholm: city, SoRAD.

Michopoulos, I., Douzenis, A., Kalkavoura, C. Christodoulou, C., Michalopoulou, P., Kalemi, G.. . . Lykouras, L. (2008). Hospital Anxiety and Depression Scale (HADS): validation in a Greek general hospital sample. *Annals of General Psychiatry, 7* (1), 4.

Newman, MG, Szkodny, LE, Llera, SJ, & Przeworski, A. (2010). A review of technology-assisted self-help and minimal contact therapies for drug and alcohol abuse and smoking addiction: Is human contact Necessary for therapeutic efficacy? *Clin Psychol Rev., 31* (2011), 178-186.

O'Carroll, RE, Smith, K., Couston, M., Cossar, JA, & Hayes, PC (2000). A comparison of the WHOQOL-100 and the WHOQOL-BREF detecting the change in quality of life Following liver transplantation. *Quality of Life Research, 9* (1), 121-124. doi: 10.1023 / A: 1008901320492

Postel, MG (2011). *Well connected. Webbased treatment for problem drinkers. PhD dissertation.* Radboud Universiteit Nijmegen, Nijmegen, Netherlands.

Postel, MG, de Haan, HA, best Huurne, ED Becker, ES, & de Jong, CAJ (2011). Characteristics of Problem Drinkers in E-therapy versus Face-to-Face Treatment. *The American Journal of Drug and Alcohol Abuse, 37* (6), 537-542. doi: doi: 10.3109 / 00952990.2011.600388

Prochaska, J., & DiClemente, C. (1986). Toward a Comprehensive Model of Change. In N. Heather & WR Miller (Eds.), *Treating Addictive Behaviors: Processes of Change* (pp. 3-28). New York: Plenum Press.

Rabin, R., & Charro, F. d. (2001). EQ-SD: a measure of health status from the EuroQol Group. *Annals of Medicine, 33* (5), 337-343. doi: doi: 10.3109 / 07853890109002087

Roll Header, S., Heather, N., Gold, R., and Hall, W. (1992). Development of a short 'readiness to change'questionnaire For Use in brief, opportunistic intervention Among Excessive drinkers ﻿ *British Journal of Addiction, 87* (5), 743-754.

Rooke, S., Thorsteinsson, E., Karpin, A., Copeland, J., & Allsop, D. (2010). Computer-delivered intervention for alcohol and tobacco use: a meta-analysis. *Addiction, 105* (8), 1381-1390.

Saunders, JB, Aas country, OG, Babor, TF, De La Fuente, JR, & Grant, M. (1993). Development of the Alcohol Use Disorders Identification Test (AUDIT): WHO Collaborative Project on Early Detection of Persons with Harmful Alcohol Consumption - II. *Addiction,* 88, 791-804.

Saxon, L., & Wirbing, P. (2004). *Prevention: Skills training in abuse and dependence on alcohol, drugs and* medicines. Lund: Student Literature.

Sinadinovic, K. (2012). *Reaching Out: Internet-based self-assessment of problematic substance use with personalized feedback.* (PhD thesis), Karolinska Institute, Stockholm.

Sinadinovic, K. Berman, AH, Hasson, D., & Wennberg, P. (2010). Internet-based Assessment and Self-Monitoring of Problematic Alcohol and Drug Use. *Addict Behav, 35* (5), 464-470.

Sinadinovic, K., Johansson, M., Wennberg, P. & Berman, AH (2010). Treatment Issues [Questionnaire on use of other treatments During Clinical Trials on problematic substance use]. Stockholm: Karolinska Institutet.

Sinadinovic, K., Wennberg, P. & Berman, AH (2011). Population screening of risky alcohol and drug use over the Internet and Interactive Voice Response (IVR): A feasibility and psychometric study in a random sample. *Drug and Alcohol Dependence,* 114, 55-60.

Sinadinovic, K., Wennberg, P., Johansson, M., & Berman, AH (2012). Reducing problematic alcohol use with Internet-based Assessment, Personalized feedback or Cognitive Behavioral Therapy-based self-help: A randomized controlled trial. Submitted.

Sjölund, T. (2007). Effect Evaluation of Alcohol profile - an Internet-based assessment tool with personalized feedback. Stockholm: CITY.

Skevington, SM, Lotfy, M., & O'Connell, KA (2004). The World Health Organization's WHOQOL-BREF quality of life assessment: psychometric properties and results of the international field trial. A report from the WHOQOL group. [Comparative Study Validation Studies]. *Qual Life Res, 13* (2), 299-310.

Sobell LC, Sobell &, MB (1992). Timeline Follow-Back: A Technique forAssessing Self-Reported Alcohol Consumption. In R. Litten & J. Allen (Eds.), *Measuring Alcohol* Consumption. Totowa, NJ: The Humana Press, Inc..

National Board. (2012). Tobacco, alcohol, physical activity and eating habits - so can the health services support you to change unhealthy habits. Stockholm: National Board.

SOU 2011: 35th (2011). Better response to abuse and addiction. Stockholm: Ministry of Social Affairs.

WHO. (2009). Global Health Risks: mortality and burden of disease attributable to selected major Risks. (V ol. 2). Geneva: World Health Organization.

[1] This study constitutes a **pilot** study, which means that we test the treatment program's feasibility, the technical platform sustainability, and aims to get initial answers to our research questions. The study will be followed by a regular randomized, controlled trial with three arms and enough power to statistically ensure the results regarding treatment outcomes. In the future study, participants will be randmoized to TO with therapist support (possibly only asynchronous text messages based on the pilot study's results), a group that has access to self-help (either the TO as self-help or alkoholhjalpen.se), or to a control group on the waiting list for self-help or TO. The current research plan concerns only a pilot study.
