# Supplementary material for: Guided and Unguided Internet-Based Treatment for Problematic Alcohol Use – A Randomized Controlled Pilot Trial
Source: PLoS One. 2016 Jul 6;11(7):e0157817. doi: 10.1371/journal.pone.0157817 (PMC4934861; doi:10.1371/journal.pone.0157817)
Supplement: S2 Protocol — (PDF) [file pone.0157817.s003.pdf]

Internetbaserad självhjälp för problematiskt alkoholbruk - en naturalistisk studie

Anne H Berman, Magnus Johansson, Christopher Sundström,
Erik Stenlund-Gens, Kristina Sinadinovic

Bakgrund

Problematiskt alkoholbruk är idag den tredje största orsaken till sjukdom globalt sett och en av de största utmaningarna för folkhälsan då det orsakar död och funktionsnedsättningar världen över (WHO, 2009). I ett globalt perspektiv kan man tillskriva alkoholkonsumtion nästan fyra procent av alla dödsfall världen över, vilket är mer än t.ex. HIV/AIDS, våld eller tuberkulos. Konsumtion av alkohol räknas som den primära orsaken när det gäller 60 olika typer av sjukdomar och skador, och indirekt orsak när det gäller ytterligare 200.

Alkoholkonsumtionen har också ökat i Sverige sedan mitten av 1990-talet. Även om en viss minskning syns under de senaste åren är konsumtionen, enligt SoRAD:s skattningar, fortfarande knappt 20 procent högre idag jämfört med mitten av 1990-talet (H. Leifman & Ramstedt, Preliminär version 2009-11-11). I Missbruksutredningens slutbetänkande ”Bättre insatser vid missbruk och beroende” (SOU 2011:35, 2011) uppskattar utredaren att ca 700 000 personer i Sverige har ett skadligt bruk av alkohol och att 330 000 personer är beroende. När det gäller riskbruk av alkohol, som ännu inte utvecklats till skadligt bruk eller beroende, uppskattas i Missbruksutredningen att ca en miljon svenskar har ett sådant riskbruk. Statens folkhälsoinstitut år 2009 rapporterade att 18 % av männen och 10% av kvinnorna hade ett riskabelt alkoholbruk (Clevenpalm & Karlsson, 2009).

Trots att en relativt stor del av svenska befolkningen har problematiskt alkohol och drogbruk vet vi att merparten av dem inte söker behandling (Blomqvist, Cunningham, Wallander, & Collin, 2007). En kartläggning i Stockholms län visar att endast 18 % av alla med alkoholberoende i länet har kontakt med den specialiserade beroendevården (Andréasson, 2010). Det tycks finnas en stor grupp människor med behov av att förändra sina alkoholvanor, som av olika anledningar inte kommer i kontakt med beroendevården. I en attitydundersökning som genomfördes på uppdrag av Statens folkhälsoinstitut 2009 framkom att 78 % av de svarande skulle använda Internet för att skaffa sig information om alkohol och andra droger och i fokusgruppsintervjuer med riskkonsumenter av alkohol framkom att den hjälp man i första hand vill ha för sin problematiska användning utgörs av vederhäftig information, gärna i form av webbaserade test. Studier på svenska Internetbaserade

självtest för problematisk alkohol- och droganvändning visar att dessa utgör ett effektivt sätt att nå både dem med problematiskt bruk och de grupper som i mindre utsträckning nås av vården, som t.ex. kvinnor och unga vuxna (Sinadinovic, Berman, Hasson, & Wennberg, 2010; Sjölund, 2007).

Ur ett genusperspektiv har alkoholkonsumtionen i Sverige vid en jämförelse med förhållandena under 1990-talet ökat snabbare bland kvinnor än bland män (CAN, 2009) och en befolkningsscreening från 2008 visade inte några större skillnader i förekomsten av problematisk alkoholanvändning mellan kvinnor och män, då totalt 20 % av kvinnorna och 22 % av männen hade ett problematiskt bruk av alkohol. Den problematiska användningen av droger visade sig vara vanligare bland kvinnor – 3,8 % – än frekvensen 1,8 % bland män (Sinadinovic, Wennberg, & Berman, 2011), eventuellt på grund av högre förekomst av läkemedelsmissbruk bland kvinnorna. Könsfördelningen inom den specialiserade beroendevården i Stockholms län visar dock att endast 33 % av patienterna under 2010 var kvinnor och 67 % män (A. Leifman, 2011). Liknande siffror rapporteras i Nederländerna (Postel, de Haan, ter Huurne, Becker, & de Jong, 2011). Detta tyder på att det är särskilt svårt för beroendevården att nå kvinnor med problematiskt bruk av alkohol och droger. Samtidigt har tidigare forskning visat att det just är kvinnor som särskilt tilltalas av Internetbaserade interventioner och att när det kommer till användningen av sådana tjänster är könsfördelningen minst 50 % kvinnor (Koski-Jännes, Cunningham, Tolonen, & Bothas, 2007; Postel et al., 2011; Sinadinovic, Berman, et al., 2010). Att leverera interventioner för problematisk alkohol- och droganvändning via Internet bör vara lämpligt för svenska förhållanden då 85 % av svenska befolkningen använder Internet och Sverige är ett av de länderna i världen där Internetanvändningen är högst och mest spridd i samhället; dessutom visar aktuell svensk forskning att internetbaserade interventioner för problematiskt alkohol- och drogbruk tilltalar användare i den svenska befolkningen (Sinadinovic, 2012). En kanadensisk studie har också visat att majoriteten av människor med problematisk användning av alkohol och/eller droger använder Internet, till och med i högre utsträckning än de utan sådana problematiska vanor (Cunningham, Selby, Kypri, & Humphreys, 2006).

Hittills har behandling för problematiskt alkoholbruk via Internet undersökts i Sverige enbart i självhjälpsformat. Individer med alkoholproblem randomiserades till 1) en interventionsgrupp med internetbaserad bedömning med automatiserad återkoppling (eScreen.se), 2) en interventionsgrupp med internetbaserad självhjälp (alkoholhjalpen.se) eller till 3) en kontrollgrupp som endast fick fylla i bedömningsformulär. Ingen kontakt med behandlare förekom i någon av grupperna. Alkoholkonsumtionen och alkoholrelaterade problem minskade bland studiedeltagarna från alla tre

grupperna under de första 3 månaderna de deltog i studien och nivåerna förblev därefter stabila i ytterligare 9 månader. Den korta interventionen eScreen.se visade sig vara lika effektiv för att minska alkoholanvändningen som enbart besvarandet av bedömningsfrågorna. För dem som använt eScreen.se hade en större andel gått vidare till att samtala med någon om sina problem. Visst stöd hittades för att användningen av den mer omfattande interventionen Alkoholhjälpen var något effektivare än eScreen.se och enbart besvarandet av alkoholfrågor för detta syfte men dessa resultat är inte entydiga då flera analyser pekar på att det inte fanns några effektskillnader. (Sinadinovic, Wennberg, Johansson, & Berman, 2013). Resultaten pekar på samstämmighet med de överlag positiva resultaten från studier som ingått i översiktsartiklar och meta-analyser (Newman, Szkodny, Llera, & Przeworski, 2010; Rooke, Thorsteinsson, Karpin, Copeland, & Allsop, 2010). Vår forskargrupp har mellan december 2012 och mars 2013 genomfört en pilotstudie där vi erbjudit en internetbaserad självhjälpsintervention, eChange, med tillägg av behandlarstöd, för att testa vad tillgången till en personlig behandlare – som ger rådgivning via skriftliga meddelanden eller chatt – kan tillföra behandlingen utöver enbart självhjälp.

Denna projektplan avser en naturalistisk studie av den internetbaserade interventionen eChange, där programmet erbjuds enbart som självhjälp, d v s utan behandlarstöd. Vi har tidigare genomfört en studie där innehållet i självhjälpssajten *Alkoholhjalpens självhjälp* ingått i en arm av en randomiserad, kontrollerad studie (Sinadinovic et al., 2013). eChange är uppbyggd på ett annat sätt än *Alkoholhjälpens självhjälp* då eChange erbjuder ett strukturerat återfallspreventionsprogram. Vi vill därför studera eChange särskilt. Ytterligare ett motiv är, att eChange redan studerats i Holland med mycket goda resultat, såväl med som utan behandlarstöd.

Alkoholhjalpen.se används sedan december 2012 som en portal för en kommande satsning på en s k stepped care modell för internetbaserade interventioner kring alkoholproblem. För att skilja mellan portalen *Alkoholhjalpen.se* och innehållet i det självhjälpsprogrammet som testats i Sinadinovic et al., 2013, kallas här självhjälpsprogrammet för *Alkoholhjalpens självhjälp.* Det kan vara svårt för en utomstående att förstå skillnaderna mellan *eChange* och *Alkoholhjalpens självhjälp.* Nedanstående tabell ger därför en översikt över skillnaderna mellan *eChange* och *Alkoholhjalpens självhjälp,* inklusive framtida forskningsplaneringen.

**Innehåll**	*eChange*	*Alkoholhjälpens självhjälp*
**Källa**	Jellinekkliniken, Nederländerna, översatt och anpassad för svenska förhållanden av forskargruppen, enligt avtal med Jellinek.	Alkoholkommittén i Sverige, genom Magnus Johansson, Sven Andreasson med flera.

**Syfte**	Återfallsprevention	Reflektion och motivation till förändring. Även delar i återfallsprevention.
**Antal moduler**	8	17
**Struktur**	Strukturerat, en modul i taget blir tillgänglig för användaren	Ostrukturerat, går att välja vilken modul som helst i vilken ordning som helst
**Behandlarstöd**	Ja, via chatt eller meddelanden. Även självhjälpsvariant. Inget chattforum.	Inget behandlarstöd finns. Däremot ett modererat aktivt forum för användare med inloggningskonto.
**Forskning**	1. Holländsk pilotstudie (Blankers, Koeter, & Schippers, 2011) som visade att användare med behandlarstöd hade bättre resultat efter 6 månader än dem, med enbart självhjälp. De senare hade dock också goda resultat.<br>2. Pilotstudie (A. H. Berman, Stenlund-Gens, Sundström, Johansson, & Sinadinovic, 2012). Pågår.<br>**3. Naturalistisk studie för självhjälp som denna forskningsplan med fullständig etikansökan gäller.**<br>4. Randomiserad kontrollerad studie inom arbetslivet. Finansierad (AFA-försäkring), startas 2013 efter etikprövning.<br>5. Ordinarie randomiserad kontrollerad studie inom psykiatri och primärvård. Planeras.	Opublicerat manus under review (Sinadinovic et al., 2013).

Tabell 1. Jämförelse mellan *eChange* och *Alkoholhjälpens självhjälpsprogram*

Syfte och frågeställningar

Det övergripande syftet med denna naturalistiska studie är att studera användningen och användarna av eChange - en svensk version av ett befintligt holländskt e-hälsoprogram för självhjälp eller egenvård vid alkoholproblem. Programmet har i randomiserad kontrollerad studie visat goda resultat (Blankers et al., 2011).

Studiens primära frågeställningar är:

1. ***Vilka egenskaper har personer för vilka Internetbaserad självhjälp för alkoholproblem är effektiv?*** Effektivitet definieras som en kliniskt signifikant förändring i alkoholkonsumtion. De

egenskaper som analyseras är ålder och kön, alkoholproblemens svårighetsgrad, motivation till förändring, livskvalitet, andra psykiska problem eller konsumtion av andra droger samt annan samtidigt erhållen vård och behandling

2. ***Vilka aspekter av programmet har samband med eventuella effekter?*** De variabler som analyseras är: ”dosering” av programmet i form av antal registreringar av alkoholkonsumtion, antal genomgångna moment i programmet, antalet dagboksanteckningar samt textanalys av användarnas övningsuppgifter samt dagboksanteckningar.

Design

Som framgår ovan, är studien är en naturalistisk studie. Samtliga deltagare får tillgång till ett internetbaserat självhjälpsprogram. Mätning utifrån frågeformulär (se nedan) sker vid rekrytering till studien samt vid studiens avslut 10 veckor efter rekryteringen.

Material

Behandlingsprogrammet

Det behandlingsprogram som används i denna studie utgör en svensk översättning av ett holländskt program som visat mycket goda utfall i en randomiserad kontrollerad studie, där programmet jämfördes med en självhjälpsvariant utan behandlarstöd (Blankers et al., 2011). Behandlings-programmet innehåller 8 moduler med fokus på återfallsprevention (Saxon & Wirbing, 2004).

Utfallsmått

AUDIT (Alcohol Use Disorders Identification Test) (Bilaga 5.1)

AUDIT är ett väl belagt och mycket använt instrument för att bedöma alkoholkonsumtion (Bergman & Källmén, 2002; Saunders, Aasland, Babor, De La Fuente, & Grant, 1993). Det består av 10 frågor som besvaras från 0 till 4 och ger en slutpoäng mellan 0 och 40. Resultatet delas sedan in i riskbruk, missbruk och beroende, gränsen för riskbruk dras för män vid 8 poäng eller mer, för kvinnor vid 6 poäng eller mer. Det finns också en kort variant av instrumentet, AUDIT-C ("consumption"), som endast innehåller de tre första frågorna och används för att kartlägga aktuellt bruk. Vid vissa tillfällen kan det vara påkallat att använda någon enstaka fråga för att få en bild av någons drickande. För dessa fall är fråga 3 som berör högt episodiskt drickande den som bäst korrelerar med totalpoängen (A.H. Berman, Wennberg, & Källmén, 2012).

DUDIT (Drug Use Disorders Identification Test) (Bilaga 5.2)

DUDIT är ett frågeformulär som syftar till att ta reda på konsumtionsmönster och drogrelaterade problem av olika slag. Testet består av 11 frågor som poängsätts mellan 0 och 4, vilket ger en

slutpoäng om 44 poäng. Resultatet på testet delas in i riskbruk, missbruk och beroende. P.g.a. samhällssynen i Sverige är normerna i samråd med Socialstyrelsen satt så att det räcker med en poäng för att bruk skall klassas som riskbruk (gäller både män och kvinnor). Testet har i studier visat på god förmåga att identifiera problematiskt bruk. Precis som för AUDIT finns för DUDIT en kortare version, DUDIT-C där endast de första fyra frågorna som handlar om konsumtion ingår. Denna version av testet är dock tyvärr inte tillräckligt välstuderad för att några poänggränser skall kunna fastställas. Vad man dock kan sluta sig till är att gränsen om 1 poäng för riskbruk gäller för såväl DUDIT-C som för DUDIT. (A.H. Berman, Bergman, Palmstierna, & Schlyter, 2005; A.H. Berman et al., 2012).

TLFB (Timeline Followback) (Bilaga 5.3)

TLFB är ett instrument för att mäta konsumtion av alkohol eller droger under en viss tid och har använts framgångsrikt på perioder upp till 12 månader tillbaka (Sobell & Sobell, 1992). Det har också gjorts försök med metoden under en period av 24 månader med blandade resultat (Day, Collins, Degenhardt, Thetford, & Maher, 2004). Förfarandet enligt Sobell och Sobell (1992) går i allmänhet till så att man med hjälp av en kalender kartlägger den tid under vilken drickande skall bedömas. Förutom själva kalendern kan man använda ett skriftligt stöd för att konvertera mängd konsumerad alkohol till standardglas. Vidare söker man under tidsperioden identifiera s.k. nyckeldatum (födelsedagar, ledigheter, helger) och perioder då man druckit eller avstått alkohol. Man använder sedan nyckeldatum och perioder som s.k. ankarpunkter för att ta reda på hur mycket man drack just då. En rekommendation är att man initialt identifierar övre och undre gräns för alkoholkonsumtion under perioden så att man vet inom vilket spann man rör sig.

TLFB (Timeline Followback för 7 dagar) (Bilaga 5.3.1)

I föreliggande studie frågar vi deltagarna om deras drickande under de senaste 7 dagarna, som i studien som genomförts av Blankers et al. (2011). Värt att notera är att inget klart stöd funnits för att längre undersökningsperioder ger mer tillförlitliga resultat.

RTCQ (Readiness to Change Questionnaire) (Bilaga 5.4)

RTCQ är ett test som utvecklades för att mäta motivation till förändring (Forsberg, Ekman, Halldin, & Rönnberg, 2004; Rollnick, Heather, Gold, & Hall, 1992). Testet består av 12 frågor och följer DiClemente och Prochaskas modell om ”stadier av förändring” som beskriver processen då en person söker lösa sitt problem med beroende (Prochaska & DiClemente, 1986). I en nyligen utgiven doktorsavhandling visades att psykiatripatienter som förändrade sina alkoholvanor efter kort intervention från början övervägde att förändra sina vanor i större utsträckning än de, som inte

senare förändrade sina vanor (Gordh, 2012). Då det har rests frågor kring RTCQs förmåga att förutsäga utfall (Forsberg et al., 2004) används i denna studie även ett s k VAS-mått på förändringsmotivation.

VAS-mått på motivation (Readiness Ruler) (Bilaga 5.4.1)
Visual Analogue Scale (VAS) består av en skala där respondenten på en linje fritt placerar in sitt svar mellan två svarsalternativ, i det här fallet *”Jag är inte alls redo att förändra mina alkoholvanor”* och *”Jag är i allra högsta grad redo att förändra mina alkoholvanor”*. Höga resultat på skalan, speciellt i den övre tertiären, korrelerar med lägre nivåer av drickande vid uppföljning efter tre månader (Bertholet, Cheng, Palfai, Samet, & Saitz, 2009).

WHOQOL-BREF (Bilaga 5.5)
Ett test för att bedöma livskvalitet utvecklat av Världshälsoorganisationen WHO. Det består av 26 frågor som poängsätts från 1 till 5. Testet är en kortversion av det längre WHOQOL-100. WHOQOL-BREF korrelerar starkt med WHOQOL-100 på alla dimensionerna utom den sociala (O'Carroll, Smith, Couston, Cossar, & Hayes, 2000). Testet har använts i omfattande forskning sedan det introducerades och håller god kulturell validitet (Skevington, Lotfy, & O'Connell, 2004). Ett samarbetsavtal med WHO kring WHOQOL-BREF finns sedan 2010.

EQ-5D-5L (Bilaga 5.6)
Ett skattningsinstrument för livskvalitet som består av 5 frågor med fem svarsalternativ vardera. Till testet hör också en Visual Analogue Scale (VAS) fråga där respondenten uppmanas att markera sitt hälsotillstånd på en graderad linje mellan 0 och 100 (Rabin & Charro, 2001). Testet finns i två upplagor; dels EQ-5D-3L där varje fråga har tre svarsalternativ per fråga, dels EQ-5D-5L med fem svarsalternativ per fråga. I en senare revidering behölls de fem dimensionerna men alla svarsalternativen utökades från tre till fem (Herdman et al., 2011). Detta instrument har använts i olika kliniska populationer och kan eventuellt vara mindre känslig för livskvalite hos icke-kliniska populationer (de Willige, Wiersma, Nienhuis, & Jenner, 2005), varför såväl EQ-5D-5L och WHOQOL-BREF används i denna studie.

HADS (Hospital Anxiety and Depression Scale) (Bilaga 5.7)
HADS är ett frågeformulär som vanligen används i hälso- och sjukvården för att identifiera misstänkt ångest och depression. Formuläret består av totalt 14 frågor, 7 för ångest och depression vardera, och poängsätts från 0 till 3. Maximala poäng för ångest respektive depression är 21 och resultat på 15 poäng eller fler pekar på att vidare behandlingsåtgärder bör sättas in. Verktyget har i

studier jämförts med BDI (The Beck Depression Inventory) och STAI (Stait-Trait Anxiety Inventory) och visat på goda psykometriska egenskaper, både med avseende på korrelationen med BDI och STAI, intern konsistens och stabilitet samt test-retest (Michopoulos et al., 2008).

SRS (Session Rating Scale) (Bilaga 5.8.2)
SRS är ett verktyg för att mäta allians och administreras efter varje enskild session eller modul. Det är ett kort test där respondenten får ta ställning till i vilken utsträckning hen håller med om fyra olika påståenden; ”*Jag kände mig hörd, förstådd och respekterad.*”, ”*Vi arbetade med eller pratade om det som jag ville arbeta med och prata om.*”, ” *Sättet vi arbetade på passar mig bra.*” samt ”*Dagens samtal passade mig helt.*”. Gradering sker på en VAS-skala (Visual Analogue Scale) med ovanstående påståenden på ena sidan och samma påståenden formulerade som negationer på den andra. SRS är klart jämförbar med längre test avsedda att mäta samma sak, både när det gäller validitet och reliabilitet (Campbell & Hemsley, 2009).

Uppföljningsfrågor (Bilaga 5.9.1)
Dessa frågor behandlar på olika sätt hur deltagarna upplevde internetbehandlingen; om de saknade kontakt med behandlare, om de upplevde behandlingen som effektiv samt om de skulle rekommendera den till någon annan. Svaren är ömsom graderade, ömsom formulerade som ”ja, ”nej” och ”vet ej” (Postel, 2011).

Behandlingsfrågor (Bilaga 5.10)
Detta korta frågeformulär som utvecklats av forskargruppen innehåller tre frågor som avser fånga in om studiedeltagaren använt sig av andra åtgärder förutom internetbehandlingen för att hantera sitt problem, i det här fallet problematisk alkoholkonsumtion. Frågorna handlar om medicinering och om personen haft kontakt med någon annan hjälpinstans – såväl självhjälp, telefonrådgivning eller sedvanlig vård. Genom att kontrollera för övriga faktorer blir det lättare att bedöma vilken effekt den genomförda behandlingen haft (Sinadinovic, 2012; Sinadinovic, Johansson, Wennberg, & Berman, 2010).

Deltagare

I likhet med tjänsten *eScreen.se* där användarna sedan 2007 ingår i en pågående naturalistisk studie om vilken data publicerades 2010 (Sinadinovic 2010), vill vi låta denna studie pågå under en obestämbar tid framöver. All information och informerat samtycke kommer att ske på internetbaserad plattform i likhet med tidigare studier som vi genomfört. Individer som är 18 år eller äldre med minst riskbruk av alkohol (≥6 p för kvinnor och ≥8 p för män) enligt Alcohol Use

Disorders Identification Test (AUDIT) inkluderas i studien. Vidare kommer vi att informera deltagarna om att det är viktigt att de har för avsikt att finnas tillgängliga via internet för studien inom de närmaste 10 veckorna som studien pågår.

Personer under 18 år och/eller utan riskbruk av alkohol exkluderas.

Personer som får över 15 på skattningar av ångest respektive depression på HADS, de som får ett resultat som är två standardavvikelser eller mer under genomsnittet på något av de instrument som mäter livskvalitet (WHOQOL-BREF och EQ-5D) får delta i studien men rekommenderas efter 10 veckor vid kvarvarande allvarlig problematik att söka psykiatrisk hjälp. Deltagare som i slutet av studien har poäng högre än 20 på AUDIT kommer att få rekommendation att vända sig till den specialiserade beroendevården eller missbruksvård som tillhandahålls genom socialtjänsten. Deltagare som enligt Drug Use Disorders Identification Test (DUDIT) har ett aktivt drogbruk parallellt med alkoholbruket kommer att få delta i studien men vid kvarvarande drogbruk i slutet av studien kommer de att få rekommendation att som ovan vända sig till den specialiserade beroendevården eller socialtjänst för fortsatt hjälp. Personliga råd om detta kan ges om personen kontaktar forskargruppen. Kontaktuppgifter kommer att finnas tillgängliga vid inloggning på det personliga användarkontot genom behandlingssajten.

Att notera när det gäller droger är att vi förväntar oss att en mindre andel av studiedeltagarna kommer att visa tecken på ett problematiskt drogbruk parallellt med problematiskt alkoholbruk. En befolkningsundersökning av narkotikaproblem från år 2008, där DUDIT-formuläret användes, visar nämligen på riskbruk av droger hos 2% av svenska befolkningen och skadligt bruk hos 2,8% (Sinadinovic et al., 2011).

Procedur

Rekrytering

Rekrytering till studien kommer att ske via en annons som publiceras på portalen *Alkoholhjälpen.se*. De som visar intresse att delta får ta del av informerat samtycke (se bilaga 4.1) samt ange kön och ålder och fylla i AUDIT-formuläret.

Om inklusionskriterierna för ålder och/eller riskbruk av alkohol inte uppfylls kommer individen att informeras om att riskbruk av alkohol inte föreligger enligt svaren, och att han/hon därmed inte tillhör studiens målgrupp (bilaga 4.1.1.1).

Om inklusionskriterierna uppfylls, får deltagaren information om detta och skapar ett användarkonto (bilaga 4.1.1.2). Därefter besvaras de övriga frågeformulären (se ovan under

Material). Sedan sker en helt automatiserad randomisering till en av studiens tre armar. Deltagarna får därefter omedelbart tillgång till behandlingens första avsnitt via det egna användarkontot utifrån information anpassad efter behandlingsgrupp (bilaga 4.1.2-4). Deltagare får tillgång till en ny modul varje vecka under de första 7 veckorna. Därefter sker ett uppehåll i behandlingen i tre veckor. Sedan får deltagare tillgång till en avslutande modul.

Efter avslutad behandling fyller deltagarna i utfallsmåtten igen samt behandlingsfrågor och uppföljningsfrågor (bilagor 5.1-5.10).

Självhjälpsprogrammet

Programmet innehåller dagliga registreringar av alkoholkonsumtion och sug. Dessutom får användaren tillgång till ett nytt uppdrag varje vecka under de 6 första veckorna och ett avslutande uppdrag efter 10 veckor. Tiden efter vecka 6 och fram till vecka 10 är till för att användaren på egen hand ska testa att tillämpa lärdomarna från vecka 1-6.

vecka 1: För- och nackdelar med att dricka alkohol

vecka 2: Att bestämma mål för behandlingen

vecka 3: Att utöva självkontrollstrategier

vecka 4: Att kartlägga risksituationer

vecka 5: Att handskas med sug, känslor och socialt tryck

vecka 6: Att fastställa krisplan

vecka 10: Sammanfattning och utvärdering

Varje uppdrag innehåller en introduktions- och en övningsdel. Vissa uppdrag innehåller även ett faktablad. Deltagarna kommer att arbeta på egen hand, utan behandlarstöd under programmets gång. Dessa kommer dock vid behov att kunna ställa frågor till en support. Deltagare kommer att få en påminnelse skickad till sig via vanlig epost en gång i veckan om att ny modul finns tillgänglig.

Statistisk bearbetning

Resultaten kommer att redovisas i avidentifierad form och på statistisk gruppnivå. På så sätt kan ingen utomstående koppla viss information till en viss studiedeltagare. Deskriptiv statistik om studiedeltagarnas egenskaper utifrån frågebatteriet kommer att redovisas, liksom explorativa

analyser av korrelationer mellan studiedeltagarnas egenskaper, deras användning av programmet och utfall.

För att besvara den första primära frågeställning mäts effektivitet via kliniskt signifikanta förändringar i frekvens, kvantitet och intensivkonsumtion av alkohol, enligt TLFB. Ytterligare egenskaper mäts via de i studien ingående övriga frågor om alkoholrelaterade problem, ångest, depression, livskvalitet och hälsa. Analyserna kommer även att ta hänsyn till motivation för behandling (bilaga 5.4) samt annan behandling under studietiden (behandlingsfrågorna, bilaga 5.10).

För att besvara den andra primära frågeställningen, om vilka aspekter av programmet som har samband med eventuella effekter analyseras processmått som t ex typ och frekvens av de delar som genomförts i programmet (konsumtionsregistrering, självhjälpsövningar, dagbok). Även textanalys ingår, för att försöka identifiera textinnehåll som har samband med beteendeförändring efter genomgånget program. Dessutom kommer analyser att göras av behandlingsplattformens användbarhet (bilaga 5.9).

Slutligen kommer psykometriska analyser av kvaliteten på insamlade data att göras.

Kunskapsvinster

Svenska internetbaserade interventioner för alkohol är mycket välanvända men har endast i liten utsträckning studerats vetenskapligt. Tidigare självhjälpsinterventioner har kunnat visa effekt i form av minskad konsumtion, men det har varit oklart om innehållet i programmen har gett bättre effekter än endast screening.

Den snabba rekryteringen av studiedeltagare till den nyligen avslutade pilotstudien, med 80 deltagare på 2,5 veckor, tyder på ett stort behov i befolkningen av denna typ av hjälp. Data från ett större antal användare krävs för att få mer kunskaper om vilka användare som lyckas med att ändra sitt beteende på ett kliniskt signifikant sätt, och för att veta vilken typ av användning som hänger samman med minskad alkoholkonsumtion. Denna typ av samband har studerats i enstaka publicerade studier och kan vara av stor betydelse för vilken information som ges till potentiella användare av internetbaserad självhjälp.

Internetbaserade rekommenderas i Socialstyrelsens riktlinjer om sjukdomsförebyggande åtgärder, där webbaserad behandling för problematiskt alkoholbruk lyfts fram som en evidensbaserad åtgärd och där det betonas att vården skall vara jämlik och tillgänglig för alla (Socialstyrelsen, 2011). Socialstyrelsens riktlinjer föreskriver vidare att vården skall vara utformad enligt en s.k. stepped care-modell där lätta insatser prövas först följt av mer omfattande om den första interventionen inte

skulle ha önskad effekt. Den ökade tillgängligheten som behandling via internet potentiellt medför gör att människor som bor i glesbygd kan nås i större utsträckning. Då endast 20 % av personer med alkoholberoende söker hjälp via beroendevården kan internetbaserad behandling även nå den stora andel personer som oavsett bostadsort inte söker hjälp, eventuellt på grund av risken för stigmatisering (Blomqvist et al., 2007). Idag finns de första stegen i en sådan trappstegsmodell genom eScreen.se och Alkoholhjälpen (Sinadinovic et al., 2013) samt Alkohollinjen (Damström-Thakker, personlig kommunikation, 2012). Vår nyligen genomförda pilotstudie har tillfört data om terapeutstödd internetbehandling via eChange. Terapeutstödd internetförmedlad behandling finns i dagsläget för ett fåtal psykiatriska diagnoser, men när det gäller missbruksproblematik finns inget sådant alternativ än. På sikt tror vi att den terapeutstödda behandlingen för just missbruk och beroende av alkohol kommer att behöva erbjudas permanent för att kunna tillhandahålla ett vidare steg i modellen. För närvarande saknas dock resurser för detta.

Den aktuella studien avser en långsiktig naturalistisk studie av eChange, en alternativ självhjälpstjänst till *Alkoholhjalpens självhjälp.* Skillnaderna mellan dessa två tjänster framgår av tabell 1 ovan, där eChange erbjuder en kortare, strukturerad och redan testad intervention, jämfört med den mer omfattande, valfria strukturen och mindre beforskad *Alkoholhjalpens självhjälp.* Denna studie är ett viktigt led i arbetet för att evidensbaserade internetinterventioner som noga studerats i sitt naturliga sammanhang ska finnas tillgängliga i Sverige och erbjudas i befolkningen på ett professionellt och tillförlitligt sätt.

Referenser:

Andréasson, S. (2010). Ny utvecklingsenhet för beroendevården i Stockholms län - Redovisning av kartläggning och förslag, Preliminär version 2010-07-01. Stockholm: Statens Folkhälsoinstitut

Bergman, H., & Källmén, H. (2002). Alcohol use among Swedes and a psychometric evaluation of the Alcohol Use Disorders Identification Test. *Alcohol & Alcoholism, 37*, 245-251.

Berman, A. H., Bergman, H., Palmstierna, T., & Schlyter, F. (2005). Evaluation of the Drug Use Disorders Identification Test (DUDIT) in Criminal Justice and Detoxification Settings and in a Swedish Population Sample. *European Addiction Research, 11*, 22-31.

Berman, A. H., Stenlund-Gens, E., Sundström, C., Johansson, M., & Sinadinovic, K. (2012). *Internetbaserad behandling för problematiskt alkoholbruk - med eller utan behandlare? En randomiserad kontrollerad pilotstudie*. Karolinska Institutet, Stockholm.

Berman, A. H., Wennberg, P., & Källmén, H. (2012). *AUDIT och DUDIT – identifiera problem med alkohol och droger [AUDIT and DUDIT - identifying problematic alcohol and drug use]*. Stockholm: Gothia förlag.

Bertholet, N., Cheng, D. M., Palfai, T. P., Samet, J. H., & Saitz, R. (2009). Does readiness to change predict subsequent alcohol consumption in medical inpatients with unhealthy alcohol use? *Addictive Behaviors, 34*(8), 636-640. doi: 10.1016/j.addbeh.2009.03.034

Blankers, M., Koeter, M. W. J., & Schippers, G. (2011). Internet Therapy versus Internet Self-help versus No Treatment for Problematic Alcohol Use. *Journal of Consulting and Clinical*

Psychology, 79(3), 330–341.
Blomqvist, J., Cunningham, J., Wallander, L., & Collin, L. (2007). Att förbättra sina dryckesvanor - om olika mönster för förändring och om vad vården betyder. Stockholm: SoRAD.
Campbell, A., & Hemsley, S. (2009). Outcome Rating Scale and Session Rating Scale in psychological practice: Clinical utility of ultra-brief measures. *Clinical Psychologist, 13*(1), 1-9. doi: 10.1080/13284200802676391
CAN. (2009). Drogutvecklingen i Sverige 2009, rapport 117, http://www.can.se/documents/CAN/Rapporter/rapportserie/can-rapportserie-117-drogutvecklingen-i-sverige-2009.pdf. Stockholm: Centralförbundet för alkohol- och narkotikaupplysning.
Clevenpalm, J., & Karlsson, A.-S. (2009). Hälsa på lika villkor - Resultat från Nationella folkhälsoenkäten 2009. Östersund: Statens Folkhälsoinstitut.
Cunningham, J. A., Selby, P. L., Kypri, K., & Humphreys, K. N. (2006). Access to the Internet among drinkers, smokers and illicit drug users: Is it a barrier to the provision of interventions on the World Wide Web? *Medical informatics and the Internet in Medicine, 31*(1), 53-58.
Day, C., Collins, L., Degenhardt, L., Thetford, C., & Maher, L. (2004). Reliability of heroin users' reports of drug use behaviour using a 24 month timeline follow-back technique to assess the impact of the australian heroin shortage. *Addiction Research & Theory, 12*(5), 433-443. doi: doi:10.1080/16066350410001713231
de Willige, G. v., Wiersma, D., Nienhuis, F. J., & Jenner, J. A. (2005). Changes in quality of life in chronic psychiatric patients: A comparison between EuroQol (EQ-5D) and WHOQoL. *Quality of Life Research, 14*(2), 441-451. doi: 10.1007/s11136-004-0689-y
Forsberg, L., Ekman, S., Halldin, J., & Rönnberg, S. (2004). The readiness to change questionnaire: reliability and validity of a Swedish version and a comparison of scoring methods *British journal of health psychology*, 335-346.
Gordh, C. N. (2012). *Alcohol Use and Secondary Prevention in Psychiatric Care.* (PhD), Uppsala University, Uppsala.
Herdman, M., Gudex, C., Lloyd, A., Janssen, M. F., Kind, P., Parkin, D., . . . Badia, X. (2011). Development and preliminary testing of the new five-level version of EQ-5D (EQ-5D-5L). *Quality of Life Research, 20*(10), 1727-1736. doi: 10.1007/s11136-011-9903-x
Koski-Jännes, A., Cunningham, J., Tolonen, K., & Bothas, H. (2007). Internet-based self-assesment of drinking - 3 month follow-up data. *Addictive Behaviors, 32*, 533-542.
Leifman, A. (2011). [Utdrag ur VAL-databasen].
Leifman, H., & Ramstedt, M. (Preliminär version 2009-11-11). Svenska folkets alkoholvanor under senare år med focus på 2004-2009. Stockholm: STAD, SoRAD.
Michopoulos, I., Douzenis, A., Kalkavoura, C., Christodoulou, C., Michalopoulou, P., Kalemi, G., . . . Lykouras, L. (2008). Hospital Anxiety and Depression Scale (HADS): validation in a Greek general hospital sample. *Annals of General Psychiatry, 7*(1), 4.
Newman, M. G., Szkodny, L. E., Llera, S. J., & Przeworski, A. (2010). A review of technology-assisted self-help and minimal contact therapies for drug and alcohol abuse and smoking addiction: Is human contact necessary for therapeutic efficacy? *Clin Psychol Rev, 31*(2011), 178-186.
O'Carroll, R. E., Smith, K., Couston, M., Cossar, J. A., & Hayes, P. C. (2000). A comparison of the WHOQOL-100 and the WHOQOL-BREF in detecting change in quality of life following liver transplantation. *Quality of Life Research, 9*(1), 121-124. doi: 10.1023/a:1008901320492
Postel, M. G. (2011). *Well connected. Webbased treatment for problem drinkers. PhD dissertation.* Radboud Universiteit Nijmegen, Nijmegen, Netherlands.
Postel, M. G., de Haan, H. A., ter Huurne, E. D., Becker, E. S., & de Jong, C. A. J. (2011). Characteristics of Problem Drinkers in E-therapy versus Face-to-Face Treatment. *The American Journal of Drug and Alcohol Abuse, 37*(6), 537-542. doi:

doi:10.3109/00952990.2011.600388
Prochaska, J., & DiClemente, C. (1986). Toward a Comprehensive Model of Change. In N. Heather & W. R. Miller (Eds.), *Treating Addictive Behaviors: Processes of Change* (pp. 3-28). New York: Plenum Press.
Rabin, R., & Charro, F. d. (2001). EQ-SD: a measure of health status from the EuroQol Group. *Annals of Medicine, 33*(5), 337-343. doi: doi:10.3109/07853890109002087
Rollnick, S., Heather, N., Gold, R., & Hall, W. (1992). Development of a short 'readiness to change'questionnaire for use in brief, opportunistic interventions among excessive drinkers *British Journal of Addiction, 87*(5), 743-754.
Rooke, S., Thorsteinsson, E., Karpin, A., Copeland, J., & Allsop, D. (2010). Computer-delivered interventions for alcohol and tobacco use: a meta-analysis. *Addiction, 105*(8), 1381-1390.
Saunders, J. B., Aasland, O. G., Babor, T. F., De La Fuente, J. R., & Grant, M. (1993). Development of Alcohol Use Disorders Identification Test (AUDIT): WHO Collaborative Project on Early Detection of Persons with Harmful Alcohol Consumption - II. *Addiction, 88*, 791-804.
Saxon, L., & Wirbing, P. (2004). *Återfallsprevention: Färdighetsträning vid missbruk och beroende av alkohol, narkotika och läkemedel*. Lund: Studentlitteratur.
Sinadinovic, K. (2012). *Reaching out: Internet-based self-assessment of problematic substance use with personalized feedback.* (PhD thesis), Karolinska Institutet, Stockholm.
Sinadinovic, K., Berman, A. H., Hasson, D., & Wennberg, P. (2010). Internet-based assessment and self-monitoring of problematic alcohol and drug use. *Addict Behav, 35*(5), 464-470. doi: 10.1016/j.addbeh.2009.12.021
Sinadinovic, K., Johansson, M., Wennberg, P., & Berman, A. H. (2010). Behandlingsfrågor [Questionnaire on use of other treatments during clinical trials on problematic substance use]. Stockholm: Karolinska Institutet.
Sinadinovic, K., Wennberg, P., & Berman, A. H. (2011). Population screening of risky alcohol and drug use via Internet and Interactive Voice Response (IVR): A feasibility and psychometric study in a random sample. *Drug and Alcohol Dependence, 114*, 55-60.
Sinadinovic, K., Wennberg, P., Johansson, M., & Berman, A. H. (2013). Reducing problematic alcohol use among internet help-seekers: A randomized controlled trial comparing a screener to cognitive-behavioral self-help modules. *Submitted.*
Sjölund, T. (2007). Effektutvärdering av Alkoholprofilen – ett internetbaserat bedömningsinstrument med personlig återkoppling. Stockholm: STAD.
Skevington, S. M., Lotfy, M., & O'Connell, K. A. (2004). The World Health Organization's WHOQOL-BREF quality of life assessment: psychometric properties and results of the international field trial. A report from the WHOQOL group. [Comparative Study Validation Studies]. *Qual Life Res, 13*(2), 299-310.
Sobell, L. C., & Sobell, M. B. (1992). Timeline Follow-Back: A Technique forAssessing Self-Reported Alcohol Consumption. In R. Litten & J. Allen (Eds.), *Measuring Alcohol Consumption*. Totowa, NJ: The Humana Press, Inc. .
Socialstyrelsen. (2011). Nationella riktlinjer för sjukdomsförebyggande metoder. Tobaksbruk, riskbruk av alkohol, otillräcklig fysisk aktivitet och ohälsosamma matvanor. Stöd för styrning och ledning. Stockholm: Socialstyrelsen.
SOU 2011:35. (2011). Bättre insatser vid missbruk och beroende. Stockholm: Socialdepartementet.
WHO. (2009). Global health risks: mortality and burden of disease attributable to selected major risks. (Vol. 2). Geneva: World Health Organization.
